# A metamorphic inorganic framework that can be switched between eight single-crystalline states

**DOI:** 10.1038/ncomms14185

**Published:** 2017-02-13

**Authors:** Caihong Zhan, Jamie M. Cameron, David Gabb, Thomas Boyd, Ross S. Winter, Laia Vilà-Nadal, Scott G. Mitchell, Stefan Glatzel, Joachim Breternitz, Duncan H. Gregory, De-Liang Long, Andrew Macdonell, Leroy Cronin

**Affiliations:** 1 WestCHEM, School of Chemistry, University of Glasgow, University Avenue, Glasgow G12 8QQ, U.K

## Abstract

The design of highly flexible framework materials requires organic linkers, whereas inorganic materials are more robust but inflexible. Here, by using linkable inorganic rings made up of tungsten oxide (P_8_W_48_O_184_) building blocks, we synthesized an inorganic single crystal material that can undergo at least eight different crystal-to-crystal transformations, with gigantic crystal volume contraction and expansion changes ranging from −2,170 to +1,720 Å^3^ with no reduction in crystallinity. Not only does this material undergo the largest single crystal-to-single crystal volume transformation thus far reported (to the best of our knowledge), the system also shows conformational flexibility while maintaining robustness over several cycles in the reversible uptake and release of guest molecules switching the crystal between different metamorphic states. This material combines the robustness of inorganic materials with the flexibility of organic frameworks, thereby challenging the notion that flexible materials with robustness are mutually exclusive.

## Abstract (web summary)

Crystal engineering is a powerful process for assembling complex materials but tends to require organic building blocks, which can limit stability. Here, the authors use inorganic polyoxometalates to assemble an all-inorganic metamorphic framework that can be switched between eight distinct states.

Crystal engineering is a powerful technique for designing and assembling complex materials into crystalline lattices through the self-assembly of functional molecules^1^,^2^. The virtue of molecule-based materials is the potential for *de novo* design^3^ and post-crystallization programmability, yielding precisely engineered systems with a wide range of applications. For example, engineered crystals are central to supramolecular materials^4^, drug formulation^5^, catalysis^6^ and small-molecule sequestration^7^. However, the reliance on organic-based building blocks^8^, essential to exploit supramolecular interactions^9^, critically limits the stability and properties of the crystals^10^. In contrast to undirected self-assembly, where structures are assembled through chance combinations of smaller components, directed self-assembly is the synthesis of new materials via rational design and the careful selection of specific building blocks^11^,^12^,^13^, providing a route to the fabrication of complex functional materials. In designing our approach, we recognized the value in choosing robust building blocks with intrinsic properties that could ultimately be exploited by the final crystal structure. We decided to use polyoxometalate anions^14^, a type of molecular metal oxide, as functional building blocks for the construction of our crystal-based materials, as such assemblies have a vast number of significant intrinsic properties to exploit, such as electron storage^15^, water splitting^16^ and both acid and oxidative catalysis^17^.

Herein, we present the first reported example of what we are calling a ‘flexi-crystal'—a flexible crystalline transition metal oxide compound that is dynamically switchable between many phases and capable of performing several crystal-to-crystal transformations (starting with compound **1**, we have observed at least 8 crystal-to-crystal states connected at 11 different routes). Normally, inorganic oxide frameworks are inflexible. Although there are some inorganic oxides that exhibit small volume changes^18^,^19^,^20^,^21^,^22^. Chabazite, one of the most widespread natural zeolites and one of the first zeolites to be studied for a wide range of industrial and technological applications, showed a volume change of 0.1% after high-temperature treatment (873 K)^22^. The key building blocks of the crystal shown here are the doughnut-shaped inorganic molecular metal oxide rings, [P_8_W_48_O_184_]^40−^ ({P_8_W_48_}), connected to each other by dicationic cobalt linkers to form a lattice^23^. The molecular inorganic building block has a ring shape with a central void, which aligns in the assembled crystal to form extended pores, open to guest inclusion and exclusion (that is, water, ammonia and methanol). It is through the addition and removal of guest molecules that we witness the remarkable range of transformations for the lattice of rings (see Fig. 1 and Supplementary Movie 1). The parent compound, Li_9_K_7_W_1_Co_10_[H_2_P_8_W_48_O_186_]·132 H_2_O (**1**), is synthesized under relatively mild conditions, using a two-step approach involving the reaction of the pre-formed {P_8_W_48_} building block with Co(ClO_4_)_2_·6 H_2_O in aqueous media, and can be isolated in good yield (66%) as rectangular red crystals. Single crystal X-ray diffraction (XRD) studies reveal a one-dimensional (1D) chain structure in which a contiguous arrangement of {P_8_W_48_} rings are linked together by Co^II^ ions (see Supplementary Fig. 1). It was subsequently discovered that these crystals, through exposing them to various environmental stimuli, could assume a range of different phases and crystal forms. These forms range in the relative topology (or connectivity) of the rings and span the full range of dimensionalities, from zero to three-dimensional architectures, with fully isolated 0, 1D, two- and three-dimensional (2D and 3D) systems, shown in Figs 1 and 3.

## Results

### Crystal-to-crystal transformations

The first of these crystal-to-crystal transformations was discovered by dehydrating compound **1** through heating the crystals at 80 °C in vacuum, where they underwent a distinct colour change from red to dark purple. Unusually, the crystallinity of the material was sufficiently well-preserved during this process, to allow single crystal X-ray measurements, letting us track the formation of a new dehydrated phase, Li_9_K_7_W_1_Co_10_[H_2_P_8_W_48_O_186_]·74 H_2_O (**1**_dehydrated_). This new framework, formed by a dehydration/desorption mediated crystal-to-crystal transformation, contracted to an astonishing degree, with the cell volume decreasing from 7039.7 Å^3^ to 4869.7 Å^3^ (a total volume change of 2170 Å^3^). This transformation is, to the best of our knowledge, the largest ever reported all-inorganic oxide framework and compared with state-of-the-art ‘breathing' metal organic frameworks (MOFs) such as MIL-53(Al), which undergo a maximum volume change of 563.6 Å^3^ (ref. 24) and metal oxide zeolite RHO framework which is 552.5 Å^3^ (four times smaller than our observed volume change)^25^. On a relative scale, our material is still comparable in terms of percentage transformation (41% MIL-53, 21% zeolite RHO versus 31% for our material). It is still remarkable and sets a new precedent.

The mechanism of this large, all-inorganic crystal-to-crystal transformation can be attributed to the stability of the ring-shaped clusters and their ability to flexibly re-organize within the crystal lattice. This reorganization is facilitated by the ease with new W-O(W) and Co-O(W) bonds form and break between the linkers and inorganic rings within the crystal lattice. The resulting framework in **1**_dehydrated_ shows extensive cross-linking of the {P_8_W_48_} rings as they pack more closely in response to the loss of water, forming a tightly bound 2D columnar architecture (see Supplementary Fig. 2). Once the dark purple crystals of **1**_dehydrated_ are removed from vacuum and allowed to stand in air, a second rapid crystal-to-crystal transformation is observed with the crystals returning to a lighter pink–purple colour. Once again, the long-range order of the crystals is suitably preserved to permit crystallographic analysis. This third phase, Li_9_K_7_W_1_Co_10_[H_2_P_8_W_48_O_186_]·95 H_2_O (**1**_part.rehyd._), is found to have a partially re-expanded 2D columnar framework where the newly formed connectivity between stacked chains present in **1**_dehydrated_ is preserved, but modest rehydration of the framework results in the hydrolysis of some Co-O(W) cross-links between stacked {P_8_W_48_} rings and pendant Co^II^ cations (see Supplementary Fig. 3). This results in a re-expansion of the framework as the columnar structures reorganize, yielding a moderate increase in the measured cell volume of 219.1 Å^3^ from 4869.7 Å^3^ to 5088.8 Å^3^. This phase remains stable under normal laboratory conditions, although it is notable that both **1**_dehydrated_ and **1**_part.rehyd._ are only accessible by the post-synthetic treatment of the parent framework **1**.

**Figure 1:**
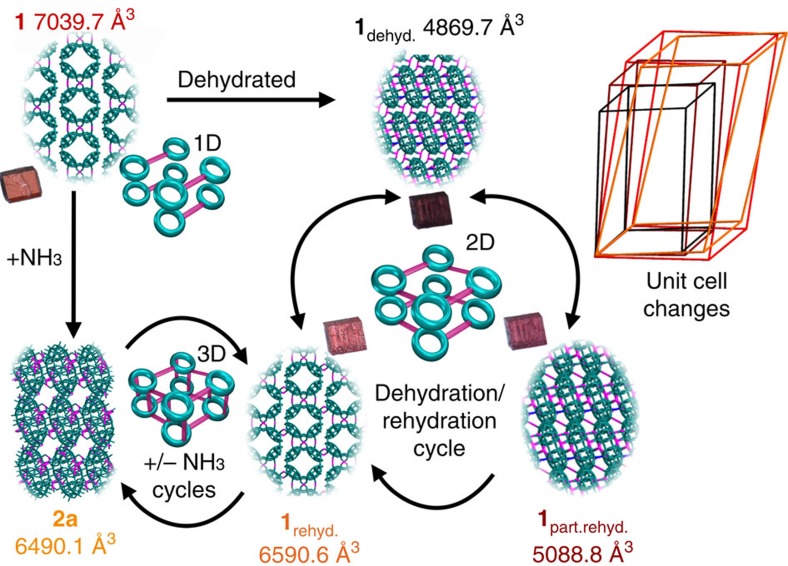
The key metamorphic crystal-to-crystal transformations observed on the addition and removal of small molecules starting from the parent compound **1**. The single-crystal structure of each framework is provided in wireframe along with the unit cell volume. The unit cells of each phase are superimposed for comparison on the RHS and colour coded **1**, red; **1**_dehyd_, black; **1**_part.rehyd._, brown; **1**_rehyd_, orange; **2a**, yellow. Structural colour code: teal, W; pink, Co; blue, W–O–W bonds.

It is also worth noting that, once the original single crystal transformation has occurred, the parent compound **1** cannot be reformed through crystal-to-crystal transformations. It is likely to be that the large volume of water present in the parent structure means the only way to form crystals of **1** is through crystallization from aqueous solution. However, under high-humidity conditions, a structure close to that of **1** is obtained. When crystals of **1**_part.rehyd._ are allowed to stand for 2 weeks under a humidified atmosphere (97% relative humidity), a third crystal transformation can be observed with the gradual return of the material to the original red colour of **1**. This fourth phase, characterized as Li_9_K_7_W_1_Co_10_[H_2_P_8_W_48_O_186_]·125 H_2_O (**1**_rehydrated_), returns to the original connectivity of the parent framework **1** with a slightly lower degree of hydration and correspondingly smaller unit cell volume—6590.6 Å^3^ (see Supplementary Fig. 4). In this instance, rehydration of the material has broken all of the cross-linking Co–O–W and W–O–W bonds between the chains along the crystallographic *a*-axis (that is, along the direction of the stacked columns) to yield a 2D sheet structure, which is topologically similar to the parent framework, **1**. Importantly, subsequent heat/vacuum treatment of this material is shown to reform **1**_dehydrated_, although it becomes increasingly difficult to preserve the crystallinity of the material and thus follow the transformation crystallographically. A summary of the transitions between compound **1**, **1**_dehydrated_, **1**_part.rehyd._ and **1**_rehydrated_ can be seen in Fig. 1.

**Figure 2:**
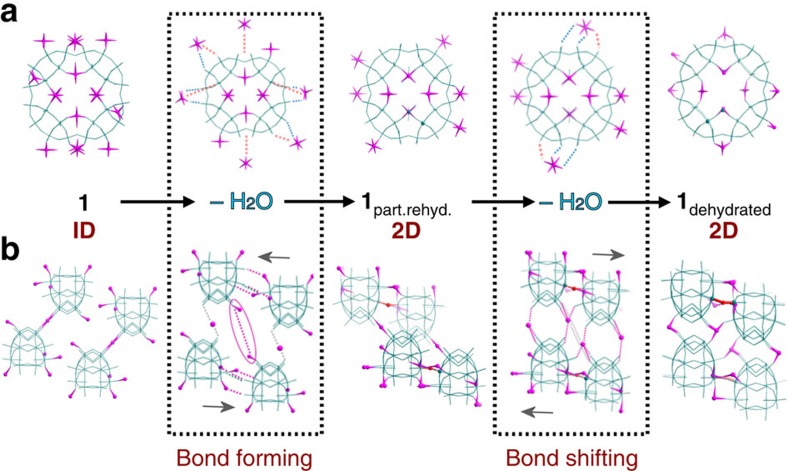
A proposed mechanism of the hydration-controlled crystal transformations of **1**. Single crystal X-ray structures show how the positional and coordinative rearrangement of the Co(II) centres in response to external stimuli (dehydration in this case) gives rise to the overall flexible properties of the material. (**a**) Face-on views of the {P_8_W_48_} cluster building unit. (**b**) Side-on views of each network represented by four linked ‘half-units'. Grey arrows are used to indicate the most probable direction along which the newly forming columns of linked {P_8_W_48_} units dislocate along the crystallographic *a* axis. Bond formation is indicated by dashed blue lines and bond-breaking by dashed orange lines, respectively. Colour code: teal, W; pink, Co; red, W–O–W bond.

Notably, the crystal-to-crystal phase transformations described above can be followed within a single crystal, isolated from the bulk product. Other multiple crystal transformations are primarily analysed via powder X-ray diffraction (PXRD), which can only follow unit cell changes and not the exact changes in molecular bonding. The single crystal analysis presented here allows for an unambiguous ‘classical' mechanistic study of the phase transformations as they occur via collection of structural ‘snapshots' of each phase, giving a deeper understanding of how our material responds to changes in its environment. Through these snapshots, we can see that the unusual ‘soft' or flexible properties of what might otherwise be considered a ‘hard' or rigid metal-oxide framework can be directly attributed to the ease with which the pendant Co^II^ cations break and reform bonds in response to external stimuli. This allows them to rearrange their position within the framework and form new structures of remarkably different topology, connectivity and size without compromising the integrity of the framework itself (Fig. 2). This is an excellent example of a concept, summarized in a recent perspective^26^, suggesting that ‘selective engineering of weaker bonds into a solid' can be used to prepare new flexible materials, while avoiding outright collapse of the framework itself.

Considering the large crystal-to-crystal transitions observed when water was added to or removed from the system, we thought it would be interesting to investigate how these frameworks would respond to the addition of other small-molecule guest species. In particular, we wanted to know whether small molecules could be used to cause further crystal-to-crystal transformations. NH_3_ was selected as an interesting model guest due to its similar size and coordination chemistry to H_2_O. Crystals of **1** were thus placed under ammonia gas (1 atmosphere, room temperature (RT)) and allowed to stand for 1 min, after which an obvious red to brown colour change occurred, suggesting rapid penetration and uptake of the NH_3_ gas into the porous framework. Remarkably, this phase transformation was also found to occur as a well-defined crystal-to-crystal transition and could thus be clearly followed crystallographically. This phase, identified as Li_9_K_7_W_1_Co_10_[H_2_P_8_W_48_O_186_]·42 H_2_O·46 NH_3_ (**2a**), has a similar 1D chain structure to **1**, but these chains are further cross-linked by exocyclic Co^II^ centres along both the *a*- and *c*-axes to form an extended 3D network (see Supplementary Fig. 5a for more detail).

**Figure 3:**
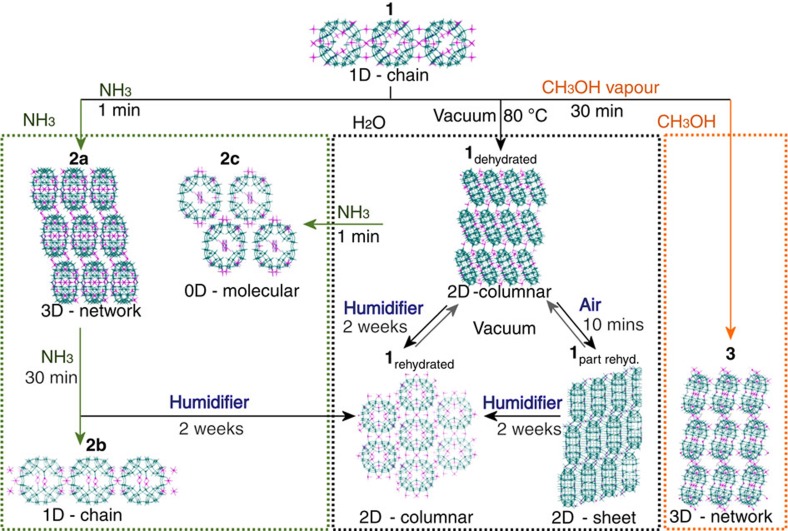
Eight crystal-forms from compound **1** accessible via 11 different transformations. Structural modification of compound **1** via guest exchange, removal or re-sorption of ammonia, water and methanol is shown. Colour code: teal, W; pink, Co.

If **1** is allowed to stand for a longer period of time (30 min) under the same NH_3_ atmosphere, it is also possible to form a second distinct crystalline phase: Li_9_K_7_W_1_Co_10_[H_2_P_8_W_48_O_186_]·35 H_2_O·44 NH_3_ (**2b**) (see Supplementary Fig. 5b for more detail). This framework reverts to a 1D-staggered chain structure, although in this case each {P_8_W_48_} ring is now linked by just a single Co^II^ cation. This feature can be further explored when crystals of **1**_dehydrated_ are subjected to NH_3_ treatment for 1 min, forming a third batch of crystalline material: Li_9_K_7_W_1_Co_10_[H_2_P_8_W_48_O_186_]·30 H_2_O·35 NH_3_ (**2c**) (see Supplementary Fig. 5c for more detail). Significantly, the pretreatment of the framework (**1** to **1**_dehydrated_) is found to have a dramatic effect on the structure of the resulting NH_3_-incorporating material. In this case, the framework structure is lost completely and the resulting compound is found to be entirely ‘molecular', comprising discrete Co^II^ substituted {P_8_W_48_} wheels. This unexpected observation is particularly noteworthy, as it suggests that the history of the framework can affect the stimuli-triggered structural response (see Fig. 3). One of the most significant aspects of this rearrangement is the behaviour of the endocyclic Co^II^ cations found in the cavity of the {P_8_W_48_} polyanion, which tend to migrate towards the centre of the cavity in response to this external stimuli, in contrast to all the hydrated species described above (see Supplementary Fig. 6).

As a point of comparison with the experiments described above, we also decided to place crystals of **1** in methanol vapour for 30 min. This yielded a crystalline phase, Li_9_K_7_W_1_Co_10_[H_2_P_8_W_48_O_186_]·40 H_2_O·18 CH_3_OH (**3**), with a new 3D-extended framework (see Supplementary Fig. 7 for full details). This does not appear to meaningfully alter the coordination modes of the endocyclic Co^II^ groups and the crystallographically identifiable MeOH is found to be exclusively located in exocyclic or interstitial positions within the crystal. This is in stark contrast to the NH_3_-treated frameworks and provides further evidence of the chemo-responsiveness of the material with respect to H_2_O, NH_3_ and MeOH.

### Differential scanning calorimetry

**Figure 4:**
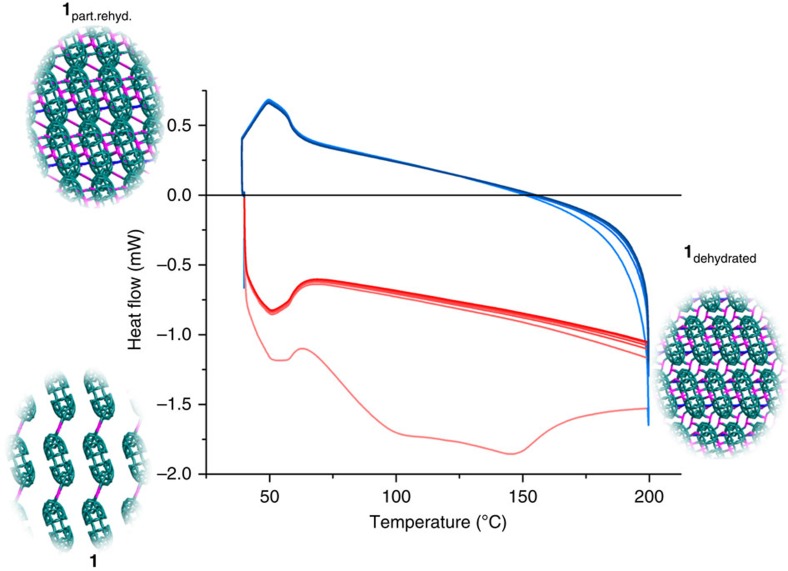
Cyclic DSC measurement of **1** cycled between 20 and 200 °C. The pale orange trace shows the initial heating cycle, involving a large two-step exothermic transition occurring during the dehydration of **1** to form **1**_dehydrated_. Subsequent cooling of the material, shown by a pale blue trace, results in a smaller endothermic transition where the material is rehydrated to form **1**_part.rehyd._, as indicated in the legend.

We can effectively cycle this all-inorganic framework material between three unique crystalline states (assuming the parent framework can never be fully recovered), simply by controlling the uptake or removal of guest H_2_O. Although it is not possible to preserve the crystallinity of the material over repeated cycles of dehydration and rehydration, thermo-analytical measurements, such as differential scanning calorimetry (DSC), allow us to monitor phase changes within the framework over a predetermined series of conditions in the absence of other structural analysis. Cyclic DSC of **1** between 20 °C and 200 °C shows a large two-step exothermic process on the initial heating cycle, indicative of the initial large phase transformation of **1** to **1**_dehydrated_ via loss of water (see Fig. 4 and Supplementary Fig. 8). On cooling, a smaller endothermic transition was observed, which corresponds to the formation of **1**_part.rehyd._ as water returns to the framework. On all subsequent heating cycles, we identified a single exothermic transition instead of the initial large two-step transition, attributed to the reformation of the fully dehydrated phase from the partly rehydrated framework. Interestingly, over six subsequent cycles of heating and cooling, the material was shown to be fully and reproducibly switchable between **1**_dehydrated_ and **1**_part.rehyd._ without any obvious signs of degradation or changes to the DSC trace. In polyoxometalate chemistry, finding crystals that can be continually dehydrated and hydrated while retaining their single-crystal nature is quite rare. This material is exceptionally robust (stable >6 cycles) and this points to its exploitable abilities.

### NH_3_ measurements

**Figure 5:**
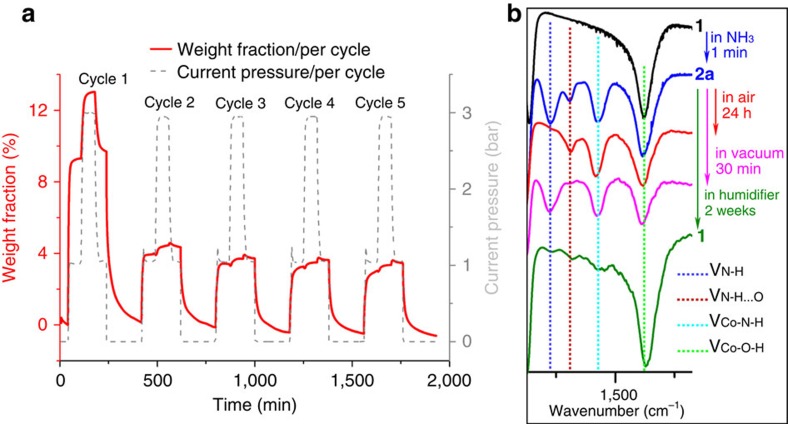
Gas uptake measurements and Fourier-transform infrared characterization of adsorbed NH_3_. (**a**) The uptake of NH_3_ gas at RT is shown for compound **1**_dehydrated_ at 1 and 3 bar pressure prior to desorption under vacuum for five cycles. (**b**) Fourier-transform infrared in the fingerprint region (1,450–1,150 cm^−1^), which shows that after the uptake of ammonia, the compound can be returned to the parent compound **1**.

The ammonia uptake was probed *in situ* and gravimetric uptake measurements were performed (see Fig. 5a and Supplementary Figs 9 and 10), which showed that the dehydrated phase **1**_dehydrated_ rapidly uptakes of 92 mg g^−1^, within 15 min at 20 °C at 1 bar, increasing to 130 mg g^−1^ at 3 bar and the uptake/release was shown to be reversible. Further, after cycle 5, the entire sample can be returned to the initial phase by rehydration and repeats the same behaviour, that is, high first uptake followed by lower uptake. The lower reversible uptake of the second to fifth cycles is attributed to the new phase formed after desorption under vacuum.

### Infrared spectroscopy

Infrared spectra of the crystals were recorded in the region 1,200–1,500 cm^−1^, to probe the chemical environment of the adsorbed NH_3_ guest molecules, allowing us to identify both coordinated and non-coordinated guests (see Fig. 5b and Supplementary Figs 11–13). When crystals of **2a** are left to stand in air for 24 h, the more weakly bound (or physically trapped) NH_3_ is lost, whereas there is no discernible change to guest species bound to the Co^II^ cations. Similarly, when crystals of **2a** are rehydrated in a humidified atmosphere, it can be shown that the chemisorbed NH_3_ is replaced by H_2_O to effectively form **1**_rehydrated_ (see Supplementary Fig. 14). The latter observation highlights the final significant feature of this material, which qualifies it as being a genuine, if unconventional, soft porous crystal: in most cases, regardless of its current phase or which guest species it has been treated with, the framework can be reproducibly returned to **1**_rehydrated_ by treatment with humidified air. The ability of the framework to return to the **1**_rehydrated_ structure can be confirmed structurally as its crystallinity is retained, as well as by a combination of powder XRD (see Supplementary Fig. 15), Fourier-transform infrared and elemental analysis (see Supplementary Fig. 16 and Supplementary Tables 1–3).

We have presented a new type of highly configurable flexi-crystal system. The phase transformations of the crystal are history dependent and programmable, with the sequence and nature of stimuli determining the final structure obtained. By using an inorganic-ring-shaped molecule with externally coordinated transition metal ions, we have shown it is possible to build up highly stable yet structurally flexible all-inorganic materials under simple solution-based conditions.

## Methods

### Materials and chemicals

All reagents and chemicals were purchased from Sigma-Aldrich and were used as provided, without further purification. K_28_Li_5_[H_7_P_8_W_48_O_184_]·92H_2_O (ref. 27) was synthesized stepwise from K_6_[P_2_W_18_O_62_]·14H_2_O (ref. 28) and K_12_[H_2_P_2_W_12_O_48_]·24H_2_O (ref. 29).

### Synthesis of Li_9_K_7_W_1_Co_10_[H_2_P_8_W_48_O_186_]·132H_2_O (**1**)

In a 50 ml round-bottomed flask, 23 ml of 2 M (aq) LiCl solution was adjusted to pH 2.23 using 1 M (aq) HCl. To this solutions, we added Co(ClO_4_)_2_·6H_2_O (635 mg, 1.74 mmol) then K_28_Li_5_[H_7_P_8_W_48_O_184_]·92H_2_O (316 mg, 0.02 mmol), resulting in the formation of a pink precipitate. The mixture was then heated at 80 °C overnight (∼20 h) with a condenser, then transferred into a conical flask. The solution was then allowed to cool to RT, with rectangular red crystals beginning to form after a period of 3 days. The solution was left to crystallize for around 1 month before the crystals were isolated, giving compound 1 in a yield of 220 mg, 13.7 mmol, 65.6 % by W. Elemental analysis calcd. (found) for H_266_O_318_Li_9_K_7_Co_10_P_8_W_49_: Co 3.79 (3.91), W 57.98 (59.81), K 1.76 (2.08), Li 0.4 (0.55), P 1.59 (1.54) (see Supplementary Table 4). Thermogravimetric analysis (TGA) measurements showed the loss of water over the temperature range of 20 to 400 °C, calcd. (found) %: 15.3 (15.12). Characteristic I.R. bands (in cm^−1^): *ν*_as_(H_2_O), 3,261 (bs); *ν*_as_(Co–O (H_2_O)), 1,622 (bs); *ν*_as_(P–O), 1,125 (s), 1,078 (s); *ν*_as_(W–O_t_), 1,012 (w), 916 (bs).

### Transformation to Li_9_K_7_W_1_Co_10_[H_2_P_8_W_48_O_186_]·74H_2_O (**1**_dehydrated_)

Fresh red-coloured single crystals of **1** were placed in a round-bottomed flask and attached to a vacuum line. The flask was evacuated for 30 min in water bath (80 °C), to ensure full dehydration of the material, and gave dark purple crystal of **1**_dehydrated_. TGA measurements showed the loss of water over the temperature range of 20 to 400 °C, calcd. (found) %: 9.2 (9.26). Characteristic I.R. bands (in cm^−1^): *ν*_as_(H_2_O), 3,261 (bm); *ν*_as_(Co–O (H_2_O)), 1,614 (bs); *ν*_as_(P–O), 1,128 (s), 1,081 (s); *ν*_as_(W–O_t_), 1,018 (w), 916 (bs).

### Transformation to Li_9_K_7_W_1_Co_10_[H_2_P_8_W_48_O_186_]·95H_2_O (**1**_part.rehyd_)

When the aforementioned round-bottomed flask was removed from the vacuum line and opened to the atmosphere, the dark purple crystals of **1**_dehydrated_ began rehydrating immediately and converted into pink–purple crystals of **1**_part.rehyd_. TGA measurements showed the loss of water over the temperature range of 20–400 °C, calcd. (found) %: 11.5 (11.51). Characteristic I.R. bands (in cm^−1^): *ν*_as_(H_2_O), 3,261 (bs); ν_as_(Co–O (H_2_O)), 1,615 (bs); *ν*_as_(P–O), 1,126 (s), 1,079 (s); *ν*_as_(W–O_t_), 1,016 (w), 916 (bs).

### Transformation to Li_9_K_7_W_1_Co_10_[H_2_P_8_W_48_O_186_]·125H_2_O (**1**_rehydrated_)

The aforementioned compound **1**_part.rehyd_ was put in a small vessel then set in a larger container with some saturated K_2_SO_4_ solution (humidity: 97%), which was sealed with a cap for 2 weeks. The pink–purple crystals of **1**_part.rehyd_ began rehydrating and converted into red-coloured crystals of **1**_rehydrated_. TGA measurements showed the loss of water over the temperature range of 20–400 °C, calcd. (found) %: 14.6 (14.6). Characteristic I.R. bands (in cm^−1^): *ν*_as_(H_2_O), 3,261 (bs); *ν*_as_(Co–O (H_2_O)), 1,621 (bs); *ν*_as_(P–O), 1,122 (s), 1,078 (s); *ν*_as_(W–O_t_), 1,011 (w), 916 (bs).

### Transformation to Li_9_K_7_W_1_Co_10_[H_2_P_8_W_48_O_186_]·42H_2_O·46NH_3_ (**2a**)

Fresh red-coloured single crystals of **1** were placed in a small vessel then put in a round-bottomed flask, which was first attached to a vacuum line for 10 s and then attached to a NH_3_ gas line (1 bar, RT) for 1 min. The red crystals converted into brown-coloured crystals of **2a**. After that, the small vessel was removed from the flask and sealed with a cap. Characteristic I.R. bands (in cm^−1^): *ν*_as_(N–H), 970 (w), 1,220 (s), 3,270 (bs); *ν*_as_(N–H…O), 1,305 (w); *ν*_as_(Co–N–H), 1,425 (s); *ν*_as_(Co–O (H_2_O)), 1,622 (bs); *ν*_as_(P–O), 1,125 (s), 1,078 (s); *ν*_as_(W–O_t_), 1,012 (w), 916 (bs).

### Transformation to Li_9_K_7_W_1_Co_10_[H_2_P_8_W_48_O_186_] 35H_2_O·44NH_3_ (**2b**)

Fresh red-coloured single crystals of **1** were placed in a small vessel then put in a round-bottomed flask, which was first attached to a vacuum line for 10 s then attached to a NH_3_ gas line (1 bar, RT) for 30 min. The red crystals converted into orange brown-coloured crystals of **2b**. After that, the small vessel was removed from the flask and sealed with a cap. Characteristic I.R. bands (in cm^−1^): *ν*_as_(N–H), 970 (w), 1,220 (s), 3,270 (bs); *ν*_as_(N–H…O), 1,305 (w); *ν*_as_(Co–N–H), 1,425 (s); *ν*_as_(Co–O (H_2_O)), 1,622 (bs); *ν*_as_(P–O), 1,125 (s), 1,078 (s); *ν*_as_(W–O_t_), 1,012 (w), 916 (bs).

### Transformation to Li_9_K_7_W_1_Co_10_[H_2_P_8_W_48_O_186_]·30H_2_O·35NH_3_ (**2c**)

The aforementioned compound **1**_dehydrated_ was placed in a small vessel then put in a round-bottomed flask, which was first attached to a vacuum line for 10 s then attached to a NH_3_ gas line (1 bar, RT) for 1 min. The purple crystals converted into reddish brown crystals of **2c**. After that, the small vessel was removed from the flask and sealed with a cap. Characteristic I.R. bands (in cm^−1^): *ν*_as_(N–H), 970 (w), 1,220 (s), 3,270 (bs); *ν*_as_(Co–N–H), 1,425 (s); *ν*_as_(Co–O (H_2_O)), 1,622 (bs); *ν*_as_(P–O), 1,125 (s), 1,078 (s); *ν*_as_(W–O_t_), 1,012 (w), 916 (bs).

### Transformation to Li_9_K_7_W_1_Co_10_[H_2_P_8_W_48_O_186_] 40H_2_O·18CH_3_OH (**3**)

Fresh red-coloured single crystals of **1** were placed in a small vessel then put in a round-bottomed flask with a small amount of methanol, which was then heated at 65 °C for 30 min. The red crystals converted into pink-coloured crystals of **3**. After that, the small vessel was removed from the flask and sealed with a cap. Characteristic I.R. bands (in cm^−1^): *ν*_as_(O–H), 3,320 (bs); *ν*_as_(C–H), 3,050–2,700 (b); *ν*_as_(C–H), 1,014 (s); *ν*_as_(Co–O (H_2_O)), 1,625 (bs); *ν*_as_(P–O), 1,125 (s), 1,078 (s); *ν*_as_(W–O_t_), 1,012 (w), 908 (bs).

### Transformation to Li_9_K_7_W_1_Co_10_[H_2_P_8_W_48_O_186_] 45H_2_O·10NH_3_ (**2a**_rehydrated_)

The aforementioned compound **2a** was put in a small vessel then set in a larger container with some saturated K_2_SO_4_ solution (humidity: 97%), which was then sealed with a cap for 2 weeks. The brown crystals of **2a** rehydrated and converted into red-coloured crystals of **2a**_rehydrated_. Characteristic I.R. bands (in cm^−1^): *ν*_as_(N–H), 1,318 (w); *ν*_as_(Co–N–H), 1,421 (s); *ν*_as_(Co–O–H), 1,616 (bs); *ν*_as_(P–O), 1,134 (s), 1,070 (s); *ν*_as_(W–O_t_), 1,011 (w), 907 (s).

### Single-crystal XRD

Suitable single crystals were selected and mounted onto the end of a thin glass fibre using Fomblin oil. Data sets for **1**, **1**_dehydrated_ and **1**_part.rehyd._ were measured at 150(2) K on an Oxford Diffraction Gemini A Ultra diffractometer, whereas crystals of **1**_rehydrated_, **2a**, **2a**_rehydrated_, **2b**, **2c** and **3** were measured on a Bruker Apex II Quasar diffractometer (λ(Mo_K*α*_)=0.71073 Å). Corrections for incident and diffracted beam absorption effects were applied using either analytical or empirical methods, respectively^30^,^31^, whereas data reduction was performed using either the CrysAlisPro or Apex2 software as supplied by the manufacturers. Final structure solution and refinement were carried out with SHELXS-97 and SHELXL-97 (or later versions) via the WinGX software suite^32^,^33^, with all structures solved by direct methods and refined using a full matrix least squares on F^2^ method. Selected details of the data collection and structural refinement of each compound can be found in Supplementary Table 5 and full details are available in the corresponding CIF files. Crystallographic data (excluding structure factors) have been deposited with the Cambridge Crystallographic Data Centre (CCDC 1429443–1429451) and may be obtained free of charge via www.ccdc.cam.ac.uk/data_request/cif.

### Powder XRD

Powder XRD patterns were collected on an Oxford Diffraction Gemini Ultra diffractometer (*λ*(CuKα)=1.5405 Å) at RT.

TGA was performed on a TGA Q500 instrument under a nitrogen atmosphere at 10 °C min^−1^. The data for relative compounds were shown in Supplementary Figs 17–20.

### DSC analysis

DSC analysis was performed on a DSC Q100 instrument.

### Fourier-transform infrared spectroscopy

Infrared spectra (4000−600 cm^−1^) were recorded on a Shimadzu FTIR 8400s spectrometer fitted with a Golden Gate ATR attachment. Intensities denoted as: s=strong, m=medium, w=weak and b=broad.

### Inductively coupled plasma optical emission spectroscopy

A minimum of 10 mg of each compound was submitted to the Institut für Festkörperforschung in Jülich, Germany, for analysis. Samples were digested in a 1:1 mixture of HNO_3_ and H_2_O_2_. A *TJA*-IRIS-Advantage spectrometer with echelle optics and CID semiconductor was used to observe across a wavelength range of 170–900 nm.

### Microanalysis

Carbon, hydrogen and nitrogen contents were determined using an EA 1110 CHNS, CE-440 Elemental Analyser. Five to 10 mg samples of the relevant compounds were submitted to the Microanalytical Service within the School of Chemistry, University of Glasgow. Carbon, hydrogen and nitrogen contents were analysed twice for each sample, with an average of the two readings presented herein.

### NH_3_ measurements

Sorption experiments were performed using a Rubotherm magnetic suspension balance setup. Details explaining this method can be found in ref. 34. In the experiment, the sample was transferred into a stainless-steel sample basket and mounted in the reaction chamber. Contact with air was reduced to a minimum (∼30 s) during the mounting process. The sample was then held in vacuum at the beginning of the experiment to remove traces of water. Depending on the experimental profile chosen, NH_3_ gas was exposed to the sample at either 1 or 3 bar pressure, with subsequent desorption steps at either 1 bar or in vacuum. Owing to the relative low pressure of the measurements, no buoyancy correction was applied. The reaction chamber was kept at 20 °C using a liquid thermostat attached to the setup.

### Data availability

The data sets generated during and analysed during the current study are available from the corresponding author on reasonable request.

## Additional information

**Accession codes:** Atomic coordinates for the reported crystal structures have been deposited with the Cambridge Structural Database under the accession codes CCDC 1429443–1429451. Full synthetic, analytical, crystallographic, as well as topological analysis is given in the Supplementary Information.

**How to cite this article**: Zhan, C. *et al*. A metamorphic inorganic framework that can be switched between eight single-crystalline states. *Nat. Commun.* **8**, 14185 doi: 10.1038/ncomms14185 (2017).

**Publisher's note**: Springer Nature remains neutral with regard to jurisdictional claims in published maps and institutional affiliations.

## Supplementary Material

Supplementary Information Supplementary Figures and Supplementary Tables

Supplementary Movie 1 Structural transformations observed upon the addition and removal of small molecules starting from the parent compound 1
