# Supplementary material for: A metamorphic inorganic framework that can be switched between eight single-crystalline states
Source: Nat Commun. 2017 Feb 13;8:14185. doi: 10.1038/ncomms14185 (PMC5316803; doi:10.1038/ncomms14185)
Supplement: Supplementary Information — Supplementary Figures and Supplementary Tables [file ncomms14185-s1.pdf]

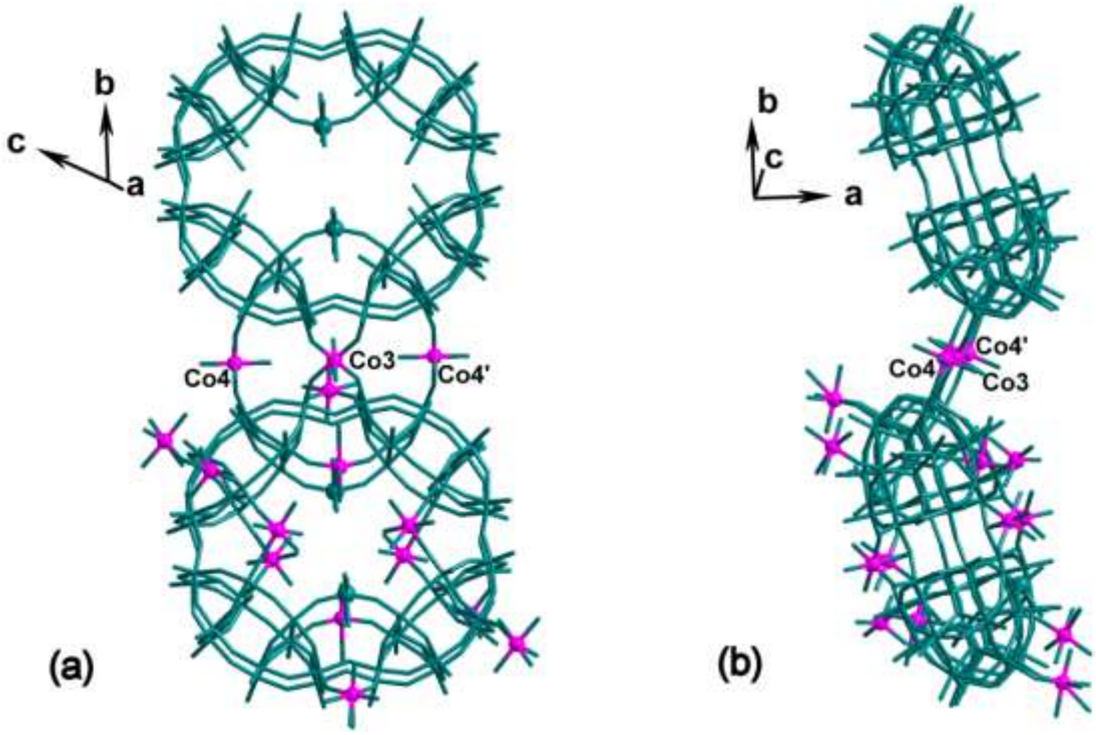

Supplementary Figure 1. Representation of the framework observed in **1** along the *a* (a) and *c* (b) axes. The {P_8W_{48}} cluster is shown in teal using a wireframe representation. The W atoms inside the cavity are represented by teal spheres and the Co atoms are represented by pink spheres. For clarity, we only show the Co atoms bound to one {P_8W_{48}} cluster. In compound 1, not all of the cobalt positions are fully occupied, so the structures are hard to describe, yet the bonding may be understood by considering each bridging Co(II) centre in turn (see Supplementary Figure 1): The first of these cobalt ions (Co3) has four μ_2 Co-O(W) bonds (avg. length = 2.108 Å) and two H_2O ligands (avg. length = 1.982 Å); on either side of these Co centers lie two equivalent Co(II) ions (Co4 and Co4′) with two μ_2 Co-O(W) bonds joining the clusters (avg. length = 1.994 Å), while the remaining four coordination sites are occupied by H_2O (avg. length = 2.091 Å). Therefore, a total of eight μ_2 Co-O(W) bonds link two adjacent {P_8W_{48}} rings (through three Co linking points) in a chain. Other Co (II) ions are attached to the {P_8W_{48}} rings independent positions and do not serve as linkers. For crystals of compound $\mathbf{1}_{\mathbf{dehydrated}}$, despite visible stress placed on the crystals during the dehydration process, the crystal integrity remains high enough for single crystal X-ray diffraction studies on the material. As not all of the Co(II) ions which decorate the {P_8W_{48}} rings in compound **1** participate in bonding between clusters, and a great deal of solvent - accessible space is occupied by water, dehydration causes a dramatic contraction of the crystal, bringing previously unconnected {P_8W_{48}} rings close enough to form additional Co-O(W) bridges. Accompanying the formation of these new transition metal-mediated linkages, the physical movement of the {P_8W_{48}} rings induced by dehydration also causes the breaking and

forming of some chain-linking Co-O(W) bonds. Overall, an additional 20 Co-O(W) bonds are formed by each cluster during this transformation, with the removal of coordinated water from Co(II) ions acting as the driving force for this structural shift. In total, twenty-eight μ_2 Co-O(W) bonds link each $\{P_8W_{48}\}$ ring in compound **1**$_{\textbf{dehydrated}}$, through eighteen individual Co(II) centers, to six neighboring clusters (see Supplementary Figure 2). Along with the formation of additional Co-O(W) bonds, the $\{P_8W_{48}\}$ rings dislocate along the crystallographic *a*-axis, moving closer together and thus forming new additional W-O(W) linkages (avg. length 1.895 Å) between previously unlinked clusters.

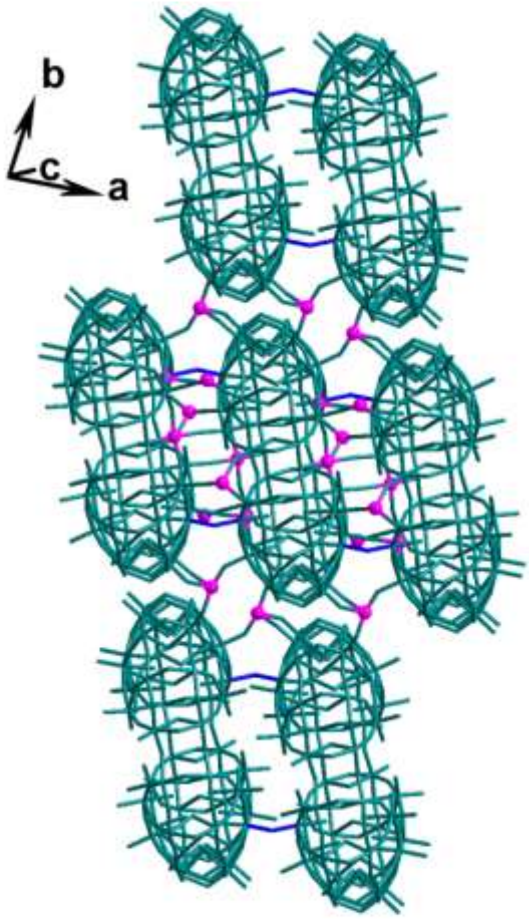

Supplementary Figure 2. Wireframe representation of the 2D, columnar framework observed in **1**$_{\mathbf{dehydrated}}$ along the *c* axis. The {P_8W_{48}} cluster is shown in wireframe (with newly formed W-O(W) bonds highlighted as blue rods). The Co atoms are represented by pink spheres. For clarity, only the Co atoms belonging to the central {P_8W_{48}} cluster are shown along with their connections to the six adjacent POM clusters. After **1**$_{\mathbf{dehydrated}}$ was removed from vacuum and allowed to stand at room temperature for 2 mins in air, the uptake of atmospheric water caused cleavage of some of the Co-O(W) bonds present in **1**$_{\mathbf{dehydrated}}$ to give a partially rehydrated phase, **1**$_{\mathbf{part.rehyd.}}$. In **1**$_{\mathbf{part.rehyd.}}$, a total of twenty-eight μ_2 Co-O(W) bonds remains the same (though this time through twenty-two individual Co ions), but they link each individual {P_8W_{48}} ring to only four neighboring clusters. The additional W-O(W) and Co-O(W) bonds which preserve the columnar structure between adjacent {P_8W_{48}} rings along the *a* axis remain intact (see Supplementary Figure 3).

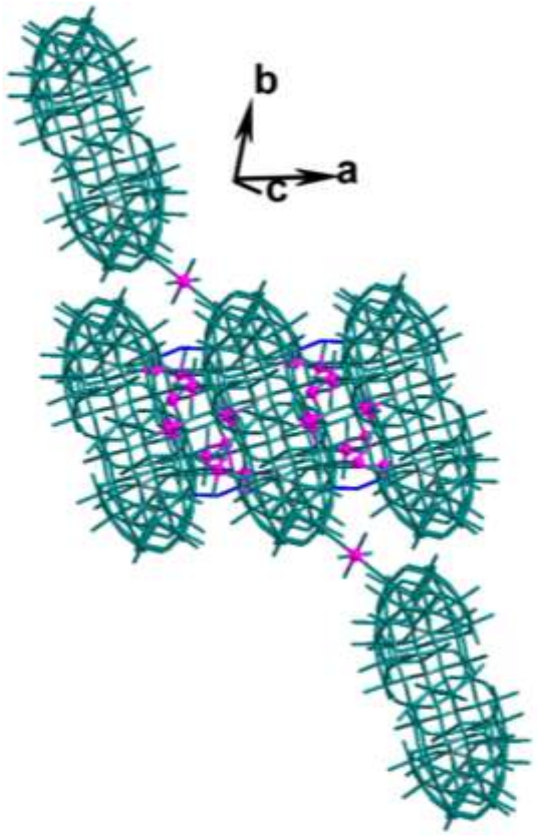

Supplementary Figure 3. Wireframe representation of the framework observed in $\mathbf{1}_{\mathbf{part.rehyd}}$. along the *c* axis. For clarity, only the Co atoms around one {P_8W_{48}} cluster are shown alongside their connection to the four adjacent POM clusters. When compound $\mathbf{1}_{\mathbf{part.rehyd}}$. was placed in a sealed container with saturated K_2SO_4 solution (humidity: 97%) for 2 weeks, it can undergo a further transformation into compound $\mathbf{1}_{\mathbf{rehydrated,}}$ which has the same fundamental chain-like structure as compound **1,** though remains further inter-connected by two pendant Co(II) linkers along the crystallographic *c-axis*, indicating that full hydrolysis of the network which would reform the topology present in the parent compound, **1**, is incomplete (see Supplementary Figure 4).

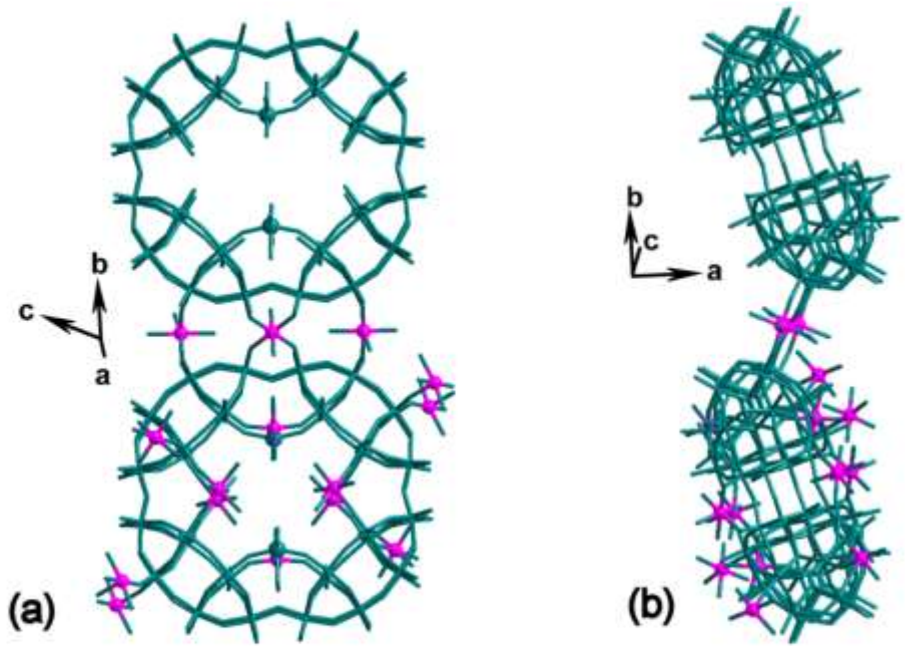

Supplementary Figure 4. Wireframe representation of the framework observed in $\mathbf{1}_{\mathbf{rehydrated}}$ along the *a* (a) and *c* (b) axes. The 49th W atom inside the cavity is represented by a teal sphere and the Co atoms are likewise represented by pink spheres. For clarity, we only show the attached Co atoms in one $\{P_8W_{48}\}$ cluster and the connection between the POM units along the crystallographic *c*-axis are omitted. From single crystal X-ray data, we can see that the packing modes of **2a**, **2b** and **2c** are remarkably similar (though they differ significantly in their connectivity/topology), but it is interesting to note that the Co cations tend to aggregate around the $\{P_8W_{48}\}$ clusters and migrate towards the middle of the central cavity within as NH_3 adsorption proceeds. As shown in Supplementary Figure 5, in compound **2a** there are three main Co(II) bridges along each chain and several Co(II) linkers sandwiched between the faces of the $\{P_8W_{48}\}$ clusters while, in compound **2b** (treated for a longer time with NH_3), two Co(II) bridges are broken and the attached Co cations migrate towards the centre of each cluster. In compound **2c** ($\mathbf{1}_{\mathbf{dehydrated}}$ in NH_3 for 1 min), all three Co(II) bridges along the *b*-axis are cleaved, moving significantly towards their parent cluster. Additionally, the W-O(W) and Co-O(W) bonds sandwiched between the faces of two adjacent $\{P_8W_{48}\}$ rings along the *a* direction were also broken, which is consistent with the result of hydration process described in the manuscript.

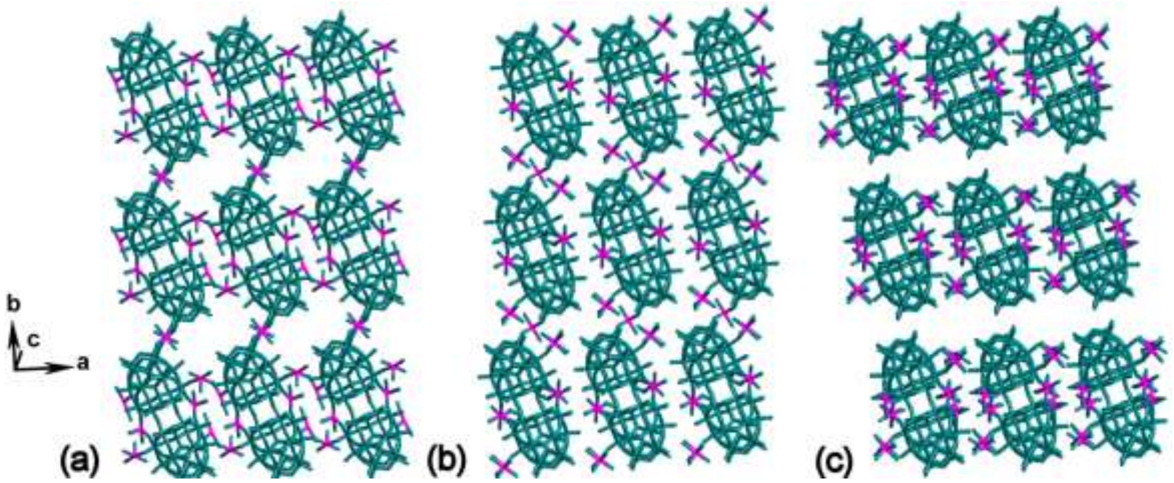

Supplementary Figure 5. Wireframe representations of the frameworks observed in $\mathbf{2_a}$ (**1** in NH_3 1 min) (a); $\mathbf{2_b}$ (**1** in NH_3 30 min) (b); and $\mathbf{2_c}$ ($\mathbf{1_{dehydrated}}$ in NH_3 1 min) (c), shown looking along the *c* axis. (Co atoms shown in pink; P, W and O atoms in teal). The framework transformations described above, the pores of the $\{P_8W_{48}\}$ motif can switch between “open” and “closed” states in response to guest accommodation. As shown in Supplementary Figure 6, during the NH_3 gas adsorption, the integrity of the $\{P_8W_{48}\}$ ring is retained, while the suspended Co cations in the pore tend to aggregate towards the center of ring, thus ‘closing’ the pores of the $\{P_8W_{48}\}$ rings. One possible explanation is that some Co-O(W) bonds in the pore are cleaved during the displacement of H_2O with NH_3 ligands, which facilitates the aggregation in the centre of the pore with a short movement of 3.75 Å. When the material is rehydrated in moist air (saturated K_2SO_4 solution, humidity: 97%), the pores are ‘opened’ again as the hydrated Co cations return to their original positions. We also note that the single crystal X-ray ‘snapshots’ obtained for **2a** and **2b** are presumably not the only transition points between **1** and **2b**, but simply the ones that could be captured by our measurements here and which represent the dynamic behaviour of the “opened” and “closed” pores in the flexible framework.

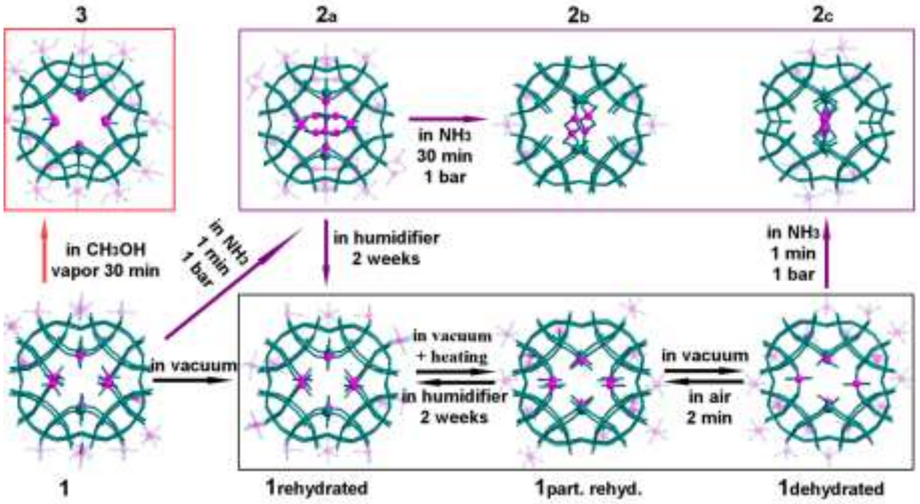

Supplementary Figure 6. Single crystal transformations of the flexible POM network, focussing specifically on the pore structure of each compound formed under different conditions (also see Fig. 5 in the manuscript for the wider framework transformations in response to the same stimuli). Interestingly, the attached Co cations in the pores of **1** do not appear to migrate during the dehydration/rehydration mediated transformations (uppermost box), while in **2a**, **2b** and **2c** (specifically, the NH_3 mediated transformations shown in the lower box), the attached cations move to different degrees under different conditions. In **2a**, **2b** and **2c**, the attached cations tend to gather in the centre of the ring and 'close' the pore as the adsorbate enters the structure, while in **3**, the larger CH_3OH molecules cannot penetrate into the cluster pore space and the endocyclic Co(II) positions are thus unaffected (*i.e.* they remain hydrated, as in the first set of transformations). What's more, **2a** can be returned to a state close to the parent compound **1** in moist air, with the endocyclic Co(II) positions returning to their original state. The $\{P_8W_{48}\}$ framework components are depicted in teal wireframe, the Co atoms as pink spheres and the 49th W atoms in the ring as teal spheres. The transparent pink spheres represent the exo-cyclic Co positions in each structure. Single crystal X-ray data shows that the packing mode of **3** is similar to compound **1,** however the connectivity of the clusters is much more extensive. The CH_3OH molecules, which are distinct from the H_2O and NH_3 guests (which themselves are crystallographically distinguishable), replace some water ligands and coordinate exclusively to the exocyclic Co cations, since the size of CH_3OH appears to be too big to effectively penetrate the central cavity of the POM units (see Supplementary Figure 7). It is worth noting that, as CH_3OH vapor diffuses into the material, some exocyclic Co cations are dehydrated and become unsaturated, so that along the *c* axis two Co cations in each $\{P_8W_{48}\}$ cluster connect the existing chain structure and form a (4, 4) 2D network (a), which are further linked by two

more Co cations on each $\{P_8W_{48}\}$ cluster along the *a* direction to yield an interconnected 3D network (b).

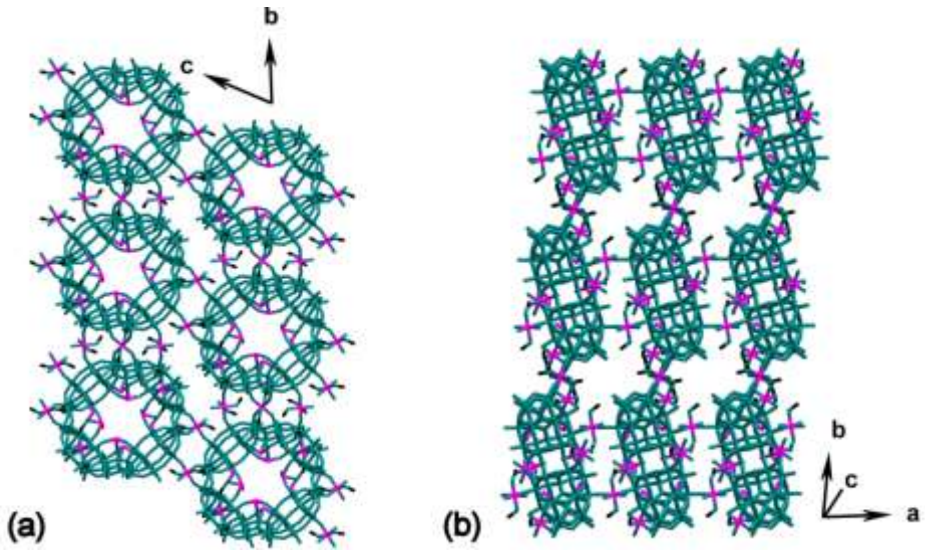

Supplementary Figure 7. Wireframe representation of the framework observed in **3** along the *a* (a) and *c* (b) axes. (Co atoms shown in pink; P, W and O atoms in teal; and C atoms in black).

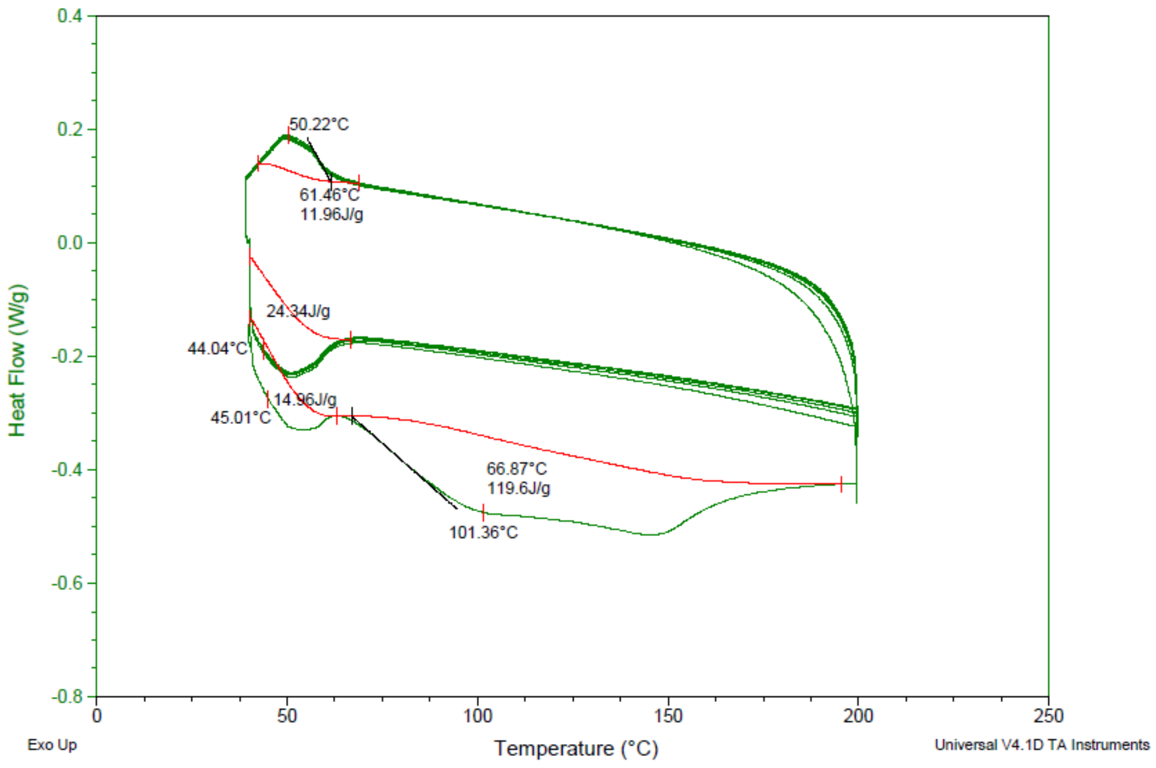

Supplementary Figure 8. Cyclic Differential scanning calorimetry of **1**. Fresh, dried crystals of **1** were heated at a rate of 5 ˚C per minute up to 200 ˚C. They were held at this temperature until their weigh remained constant. Following this the material was cooled to 40 ˚C and held at this temperature for one hour. This cycle was repeated ten times. The bottom line shows the initial heating curve, while the top line shows the cooling curve. The middle lines are all the subsequent ramps which feed into the top lines showing cooling curves. Following the initial dehydration the structure never returns to its native state. The weight loss on the first ramp (bottom line) occurs in two stages: i) removal of externally coordinated water and ii) removal of solvent water located deep inside the P_8W_{48} rings. When $\mathbf{1}_{\mathbf{part.rehyd.}}$ is heated, only the external solvent water molecules are removed (middle lines), suggesting that the inner cobalt ions are not fully rehydrated.

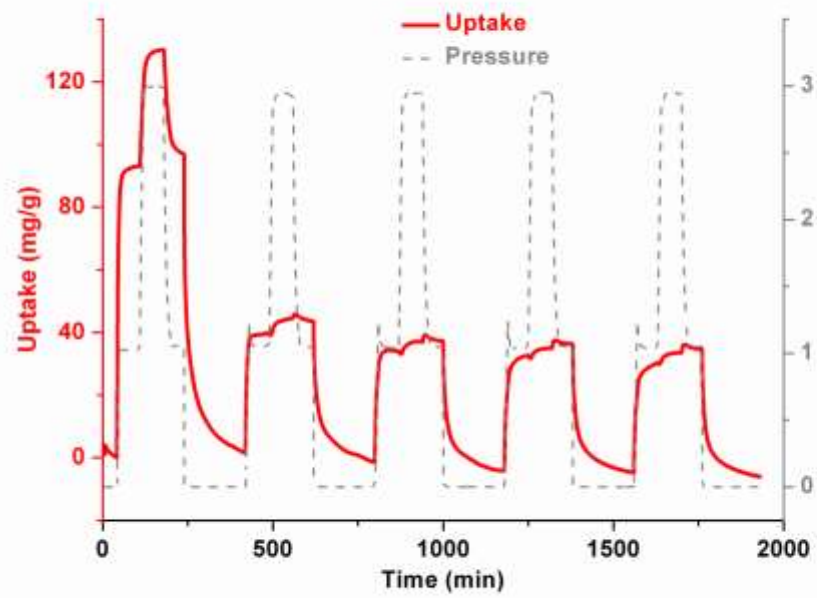

Supplementary Figure 9. The uptake of NH_3 gas at room temperature is shown for compound $\mathbf{1}_{dehydrated}$ at 1 and 3 bar pressures prior to desorption under vacuum for five cycles. The lower reversible uptake of the 2nd to 5th cycles is attributed to the new phase formed after desorption under vacuum. The coexistence of water and ammonia presents a serious corrosion threat to the magnetic suspension balance, NH_3 sorption measurements for compound $\mathbf{1}_{\mathbf{dehydrated}}$ were conducted *in situ*, while for compound **1**, we measured indirect uptake via NH_3 desorption of compound **2b**. For compound $\mathbf{1}_{\mathbf{dehydrated}}$, when it was subjected to 1 bar NH_3 gas at 20 °C, gas sorption rapidly occurs, giving a total uptake of 92 mg g^{-1}, within 15 minutes. When the pressure is increased to a maximum of 3 bar, $\mathbf{1}_{\mathbf{dehydrated}}$ is shown to have a maximum capacity of 130 mg g^{-1}, and subsequent vacuum desorption at 20 °C for 3 h is shown to remove the adsorbed guest. Notably, the material is also shown to withstand multiple reversible cycles of NH_3 adsorption and desorption from the 2nd cycle. The reduced capacity is attributed to the new phase formed after desorption under vacuum in the 1st cycle. For compound **1**, fresh red-colored crystals of **1** (259 mg) were placed in a small vessel then put in a round bottomed flask, which was first attached to a vacuum line for 10 seconds and then attached to a NH_3 gas line (1 bar, RT) for 30 min. After that, the small vessel was removed from the flask and transferred to the stainless-steel sample basket and mounted into the reaction chamber in machine. At 100˚C in vacuum, the material with NH_3 can release 33.8 mg NH_3, which means that the uptake capacity for **1** is 131 mg g^{-1} (13.5% by weight) at least.

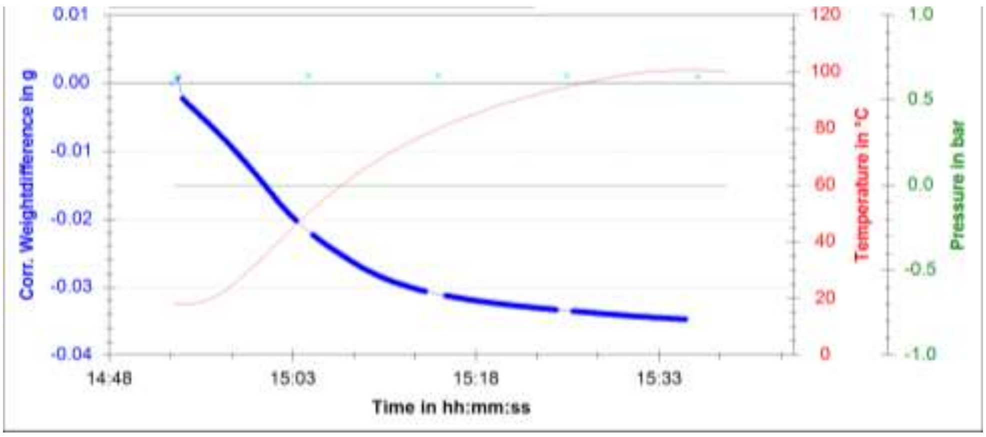

Supplementary Figure 10. Change in weight against time of NH_3 desorption of **1** at 100˚C in vacuum.

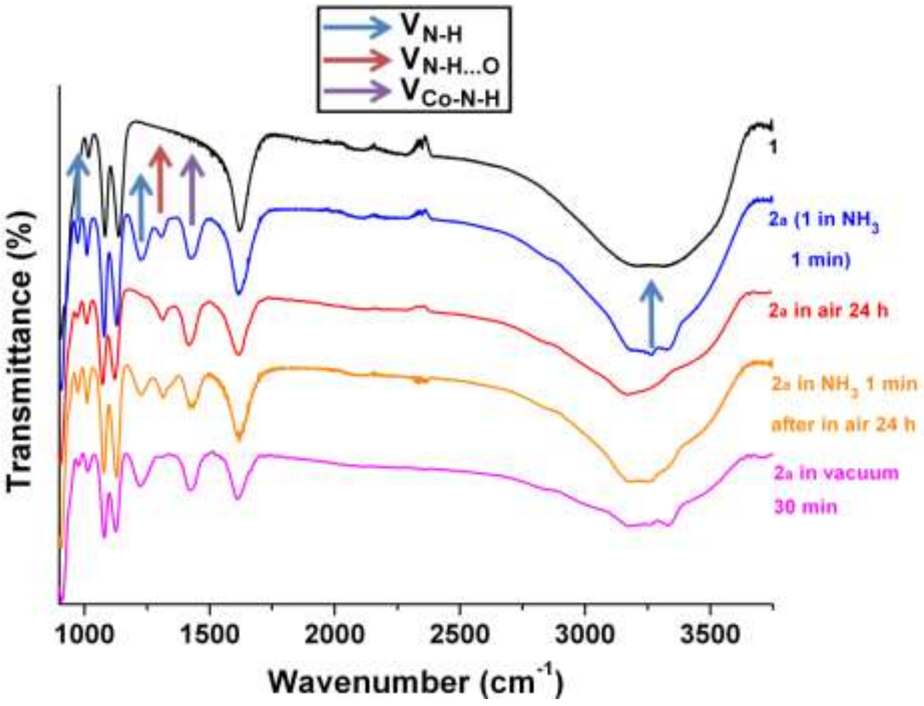

Supplementary Figure 11. Infrared spectrum of **1** and **2a** in different processes. **1** is the parent compound and **2a** is **1** treated with NH_3 for 1 min. Other Characteristic peaks: $\nu_{as}(H_2O)$, 1622 (bs); ν_{as}(P–O), 1125 (s), 1078 (s); ν_{as}(W–Ot), 1012 (w), 916 (bs). IR spectroscopy was used as a tool to probe the sites within the structures spectroscopically (see Supplementary Figure 11-13). Here, vibrations corresponding to free ammonia gas trapped in the material are indicated by the blue arrows pointing to peaks at 970, 1220, 3270 cm^{-1}. The peak indicated by the brown arrow at 1305 cm^{-1} can be assigned to the hydrogen bonded moiety N-H…O (as compound $\mathbf{1}_{\mathbf{dehydrated}}$ treated in NH_3 gas). When the ammonia gas is re-introduced again this peak re-appears. The peak shown by the violet arrow at 1425 cm^{-1} can be assigned to Co-N-H (which compares well with $Co(ClO_4)_2{\cdot}6H_2O$ when treated similarly in NH_3 gas). Obviously, therefore, the adsorption includes two distinct components. One in which the NH_3 is physically trapped in the material (either physisorbed or within a hydrogen bonding network) and can be removed by, putting the sample under vacuum or exposing it to air, and the other in which the NH_3 is directly coordinated to the available Co(II) cation sites and can only be displaced by very high levels of hydration (high-humidity air or steam). Note that in air, the hydrostatic pressure is therefore not high enough to replace all NH_3 coordinated with Co(II) (*via* ligand exchange), but if the compound is put in high humidity air or treated with steam, almost all NH_3 ligands are replaced by water molecules.

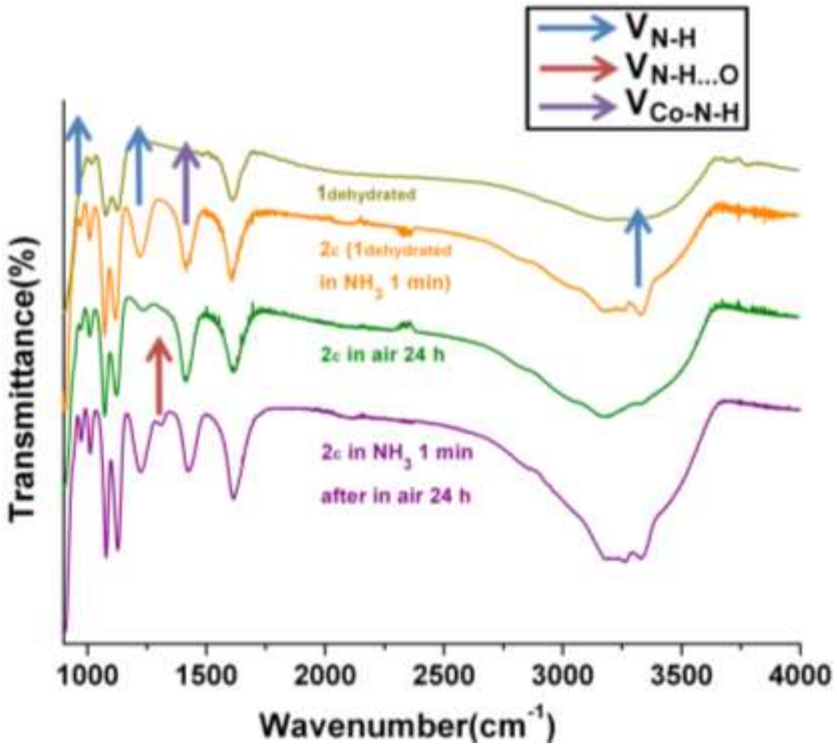

Supplementary Figure 12. Infrared spectrum of $\mathbf{1}_{\mathbf{dehydrated}}$ and **2c** in different conditions. $\mathbf{1}_{\mathbf{dehydrated}}$ is the vacuum dehydrated compound of **1** and **2c** is $\mathbf{1}_{\mathbf{dehydrated}}$ subsequently treated with NH_3 for 1 min. The peaks indicated by the blue arrows (970, 1220, 3270 cm^{-1}) stand for 'physically' trapped ammonia gas vibrations. This type of guest ammonia can be easily removed by rehydration in air. The peaks indicated by the brown arrow (1305 cm^{-1}) represents the vibration of the hydrogen bonded N-H…O moiety (compound $\mathbf{1}_{\mathbf{dehydrated}}$ in NH_3 gas does not show this peak, but when **2c** is allowed to stand in air (absorbing ambient moisture) and then put under ammonia gas again, this peak appears). The peaks indicated by the violet arrow (1425 cm^{-1}) represents the vibration of Co-N-H. Other Characteristic peaks: $v_{as}(H_2O)$, 1622 (bs); v_{as}(P–O), 1125 (s), 1078 (s); $v_{as}(W–O_t)$, 1012 (w), 916 (bs).

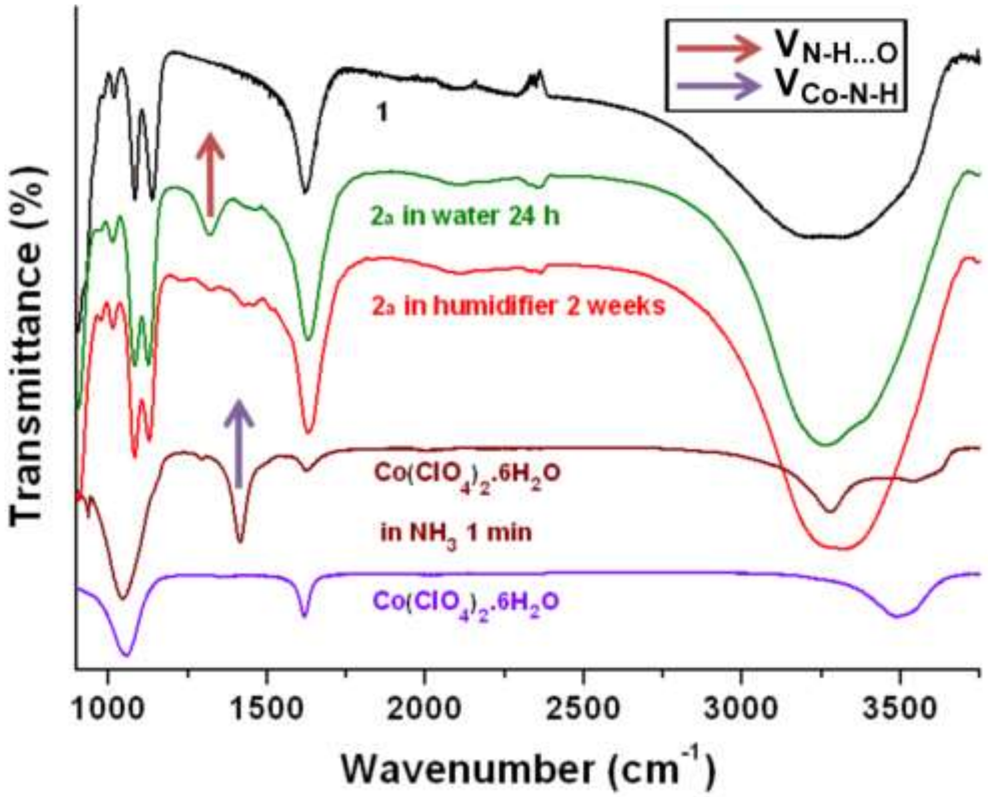

Supplementary Figure 13. Infrared spectrum of **1**, **2a** in water and the control **$Co(ClO_4)_2{\cdot}6H_2O$.** **1** is the parent compound, **2a** is **1** treated with NH_3 for 1 min. The peak indicated by the brown arrow (1305 cm^{-1}) represents the vibration of the hydrogen bonded N-H…O group. The peaks indicated by violet arrow (1425 cm^{-1}) represents the vibration of Co-N-H (as can be compared with the control compounds $Co(ClO_4)_2{\cdot}6H_2O$ and $Co(ClO_4)_2{\cdot}6NH_3$ – i.e. $Co(ClO_4)_2{\cdot}6H_2O$ treated with NH_3 gas for 1 minute). Other Characteristic peaks: $v_{as}(H_2O)$, 1622 (bs); v_{as}(P–O), 1125 (s), 1078 (s); $v_{as}(W–O_t)$, 1012 (w), 916 (bs).

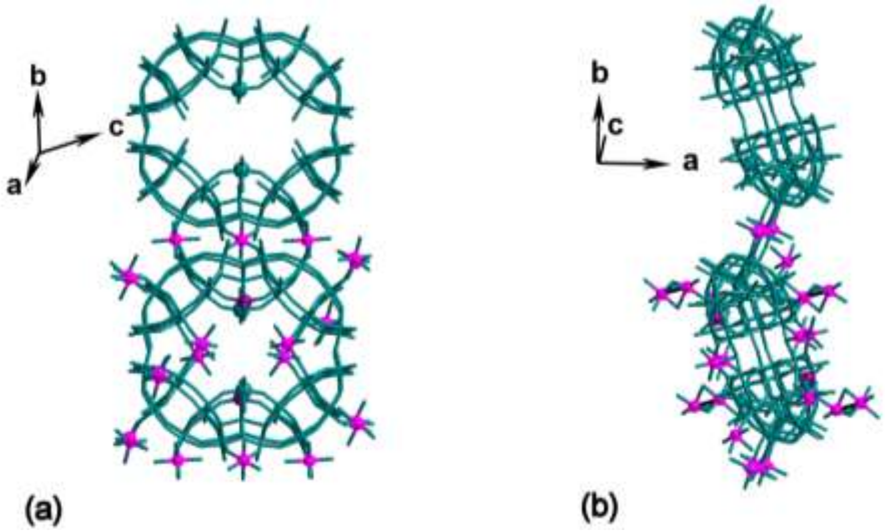

Supplementary Figure 14. If compound $\mathbf{2_a}$ is placed in a sealed container with saturated K_2SO_4 solution (humidity: 97%) for 2 weeks, it can essentially return to the same structure seen for compound $\mathbf{1_{rehydrated}}$ which has the same chain-like structure as compound **1**, though this phase ($\mathbf{2a_{rehydrated}}$) is similarly linked by additional exo-cyclic Co(II) cations in the *c*-direction, yielding a 2D topology shown above. It should be noted that there are two endocyclic, mixed Co/W positions with occupancy Co (0.8) and W (0.2). Wireframe representation of the framework observed in $\mathbf{2a_{rehydrated}}$ shown along the *a* (a) and *c* (b) axes. The mixed Co/W atom is represented by a violet sphere. For clarity, we only show the bound Co atoms for one $\{P_8W_{48}\}$ cluster and do not show the connectivity in the c-direction. Note that whilst this is a chemically distinct compound and has different unit cell parameters from compound $\mathbf{1_{rehydrated}}$, it is effectively structurally and topologically analogous which agrees with our previous observations on the rehydration of modified phases.

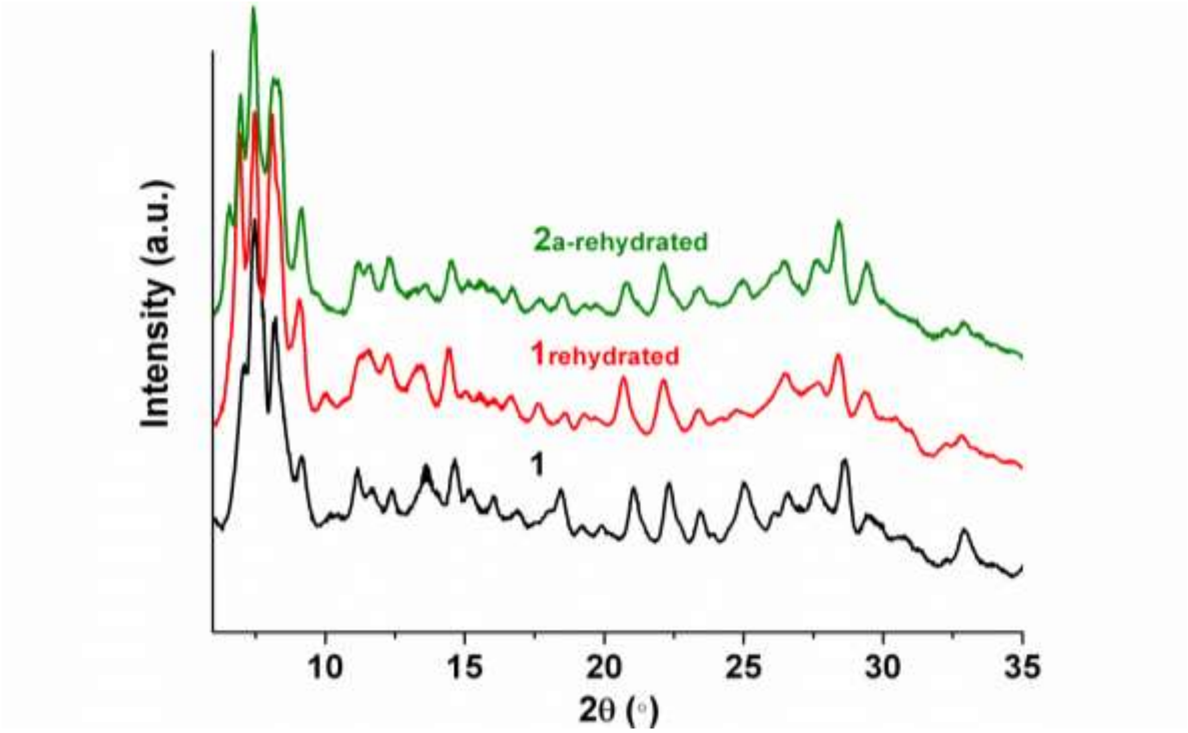

Supplementary Figure 15. Measured powder X-Ray spectra of **1**, **1**$_{\mathbf{rehydrated}}$ and **2a**$_{\mathbf{rehydrated}}$. Whilst all three datasets can be considered to be similar, the close match seen for **1**$_{\mathbf{rehydrated}}$ and **2a**$_{\mathbf{rehydrated}}$ in both the low and high angle scattering regions indicates their topological similarity, which agrees with the single-crystal X-ray studies discussed above. Whilst assignment of PXRD data for crystallographically complex POM-based materials such as these is not a trivial task, this data does help to both confirm the high degree of retained crystallinity (in the bulk material, as opposed to one single crystal) for both rehydrated phases and the structural similarity of all three phases.

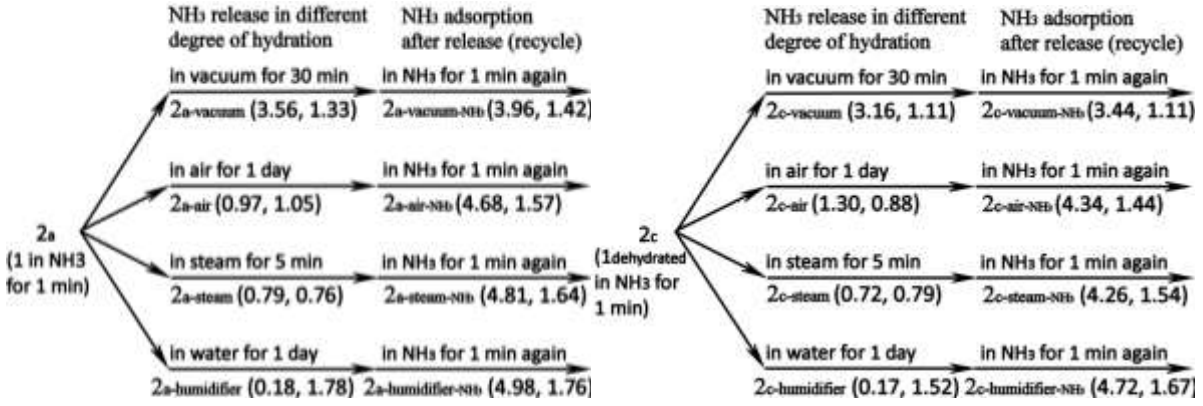

Supplementary Figure 16. Given that it is not generally possible to accurately discriminate between H_2O and NH_3 in our crystallographic measurements, microanalysis of N, H has been employed to help monitor a series of phase transitions over two cycles. Microanalysis of N and H content, respectively, for compounds **2a** and **2c** under a series of different conditions. The microanalysis measurement includes two parts. The first shows the NH_3 release upon different degrees of hydration/desolvation (in vacuum for 30 min, in air for 1 day, in steam for 5 min, in a humidifier for 1 day and 2 weeks) and the second part is the subsequent NH_3 adsorption (re-treatment with NH_3 for 1 min) after guest release (recycle). Our results show that NH_3 is better incorporated within the flexi-crystal structure under moist conditions rather than in dry conditions. As seen in Supplementary Table 1, the dehydrated material adsorbs less than the parent material (see the Microanalysis result for **2a** and **2c**), and the adsorption process is quite short (only needing 1 to 2 min, see the Microanalysis result for **2a** and **2b**). From Supplementary Table 2 and 3, It also shows that the NH_3 gas can be trapped in the material for compound **2a-vacuum** and **2c-vacuum** and subsequently released by controlling the degree of hydration (see the Microanalysis result for compound **2a-air**, **2a-steam, 2a-humidifier** and **2a-humidifier -1** or **2c-air**, **2c-steam, 2c-humidifier** and **2c-humidifier -1**). Moreover the materials can be recycled (see the Microanalysis result for compound **2a-air-NH3**, **2a-steam-NH3** and **2a-humidifier-NH3** or **2c-air-NH3**, **2c-steam-NH3** and **2c-humidifier -NH3**).

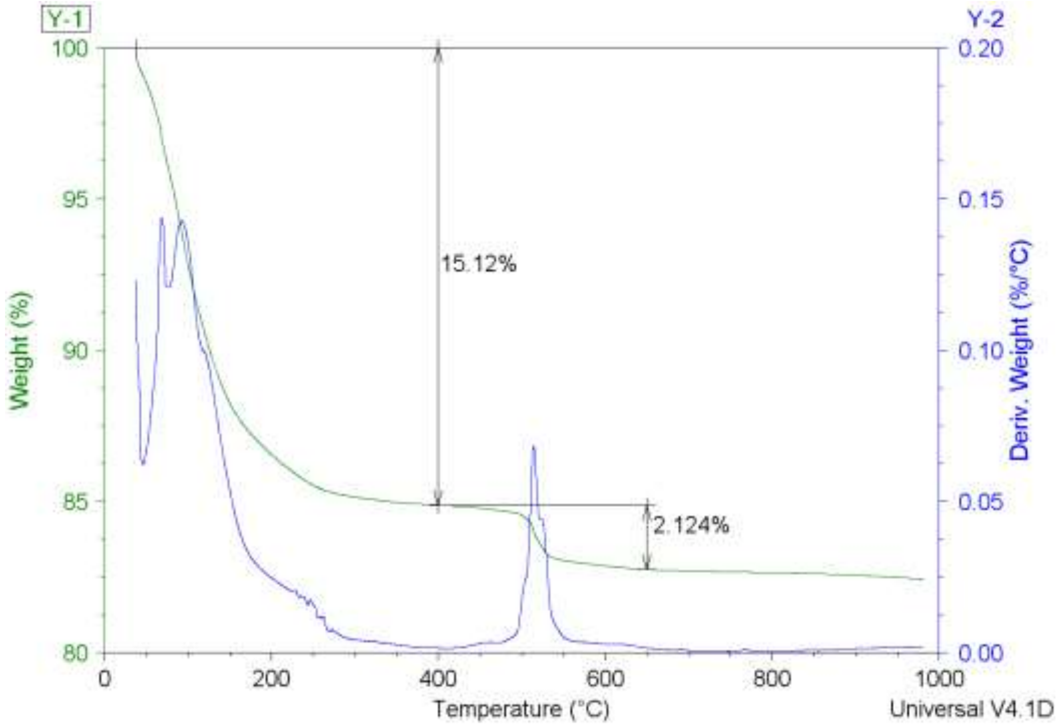

Supplementary Figure 17. Thermogravimetric analysis of **1** showing the loss of ca. 132 H_2O molecules from 0 to 400^oC, calcd. (found) %: 15.1 (15.12).

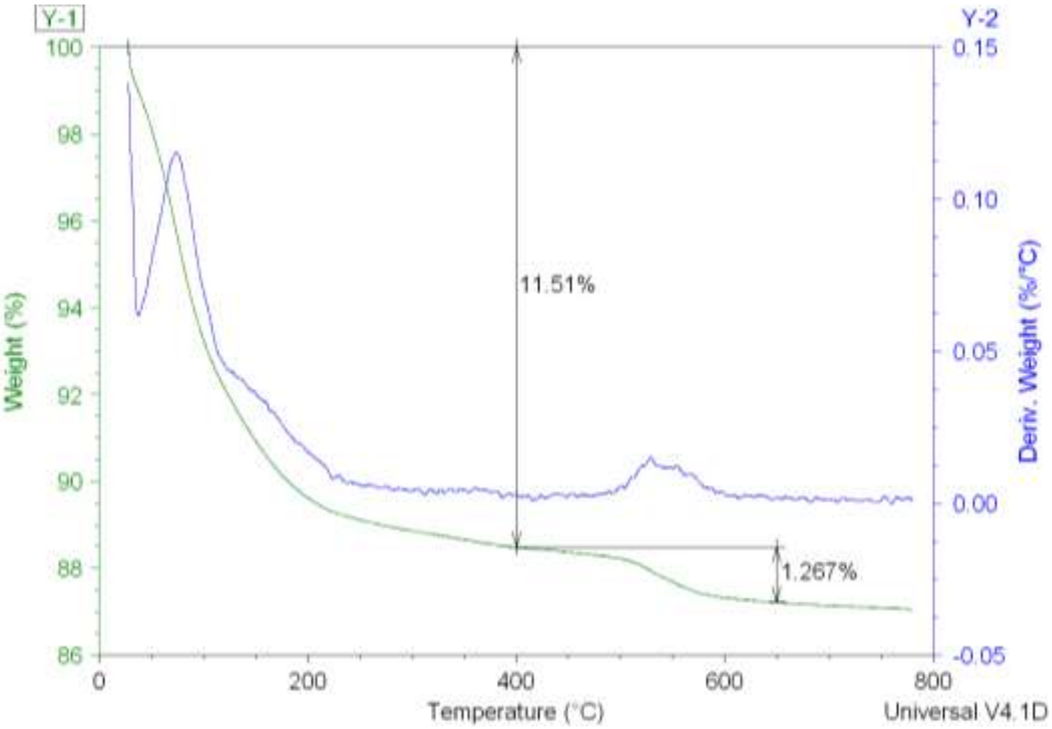

Supplementary Figure 18. Thermogravimetric analysis of $\mathbf{1}_{\mathbf{part.rehyd.}}$ showing the loss of ca. 95 H_2O molecules from 0 to 400°C, calcd. (found) %: 11.5 (11.51).

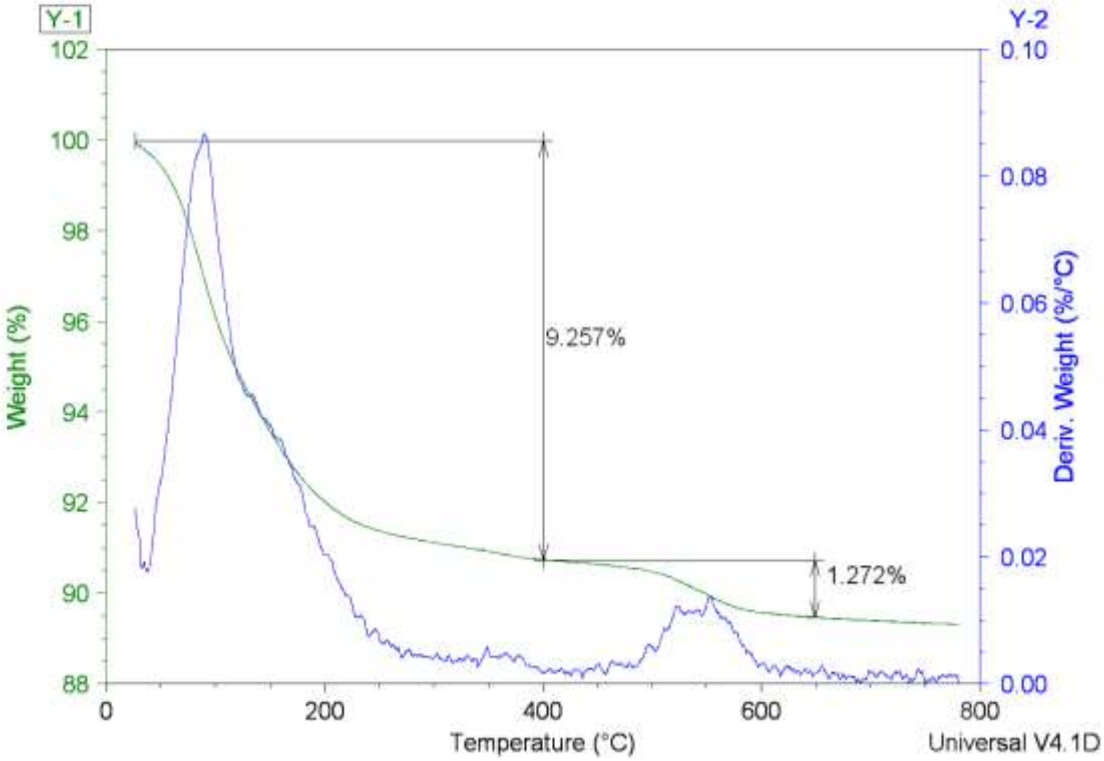

Supplementary Figure 19. Thermogravimetric analysis of $\mathbf{1}_{\mathbf{dehydrated}}$ showing the loss of ca. 74 H_2O molecules from 0 to 400°C, calcd. (found) %: 9.2 (9.26).

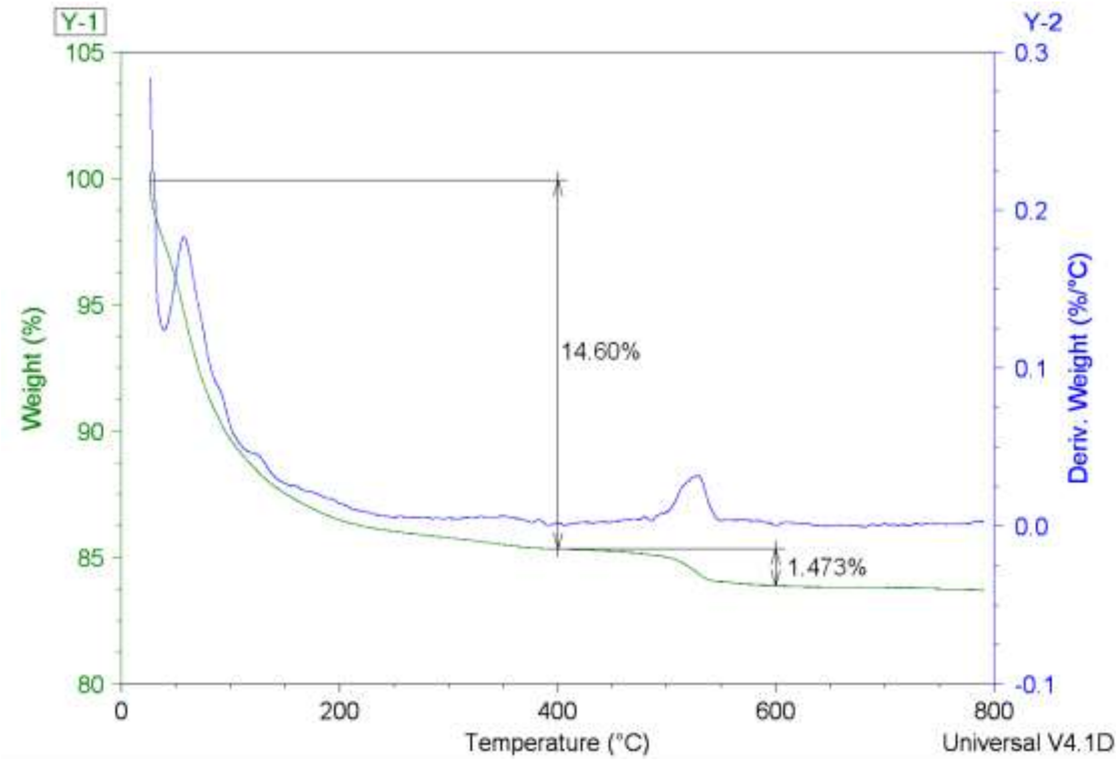

Supplementary Figure 20. Thermogravimetric analysis of $\mathbf{1}_{\mathbf{rehydrated}}$ showing the loss of ca. 125 H_2O molecules from 0 to 400°C, calcd. (found) %: 14.6 (14.6).

Supplementary Tables

Supplementary Table 1. Microanalysis of N, H of compound **1-3.**

Compound	Description	N(%)	H(%)	Formula
**1**	Parent compound	0	1.73	$Li_9K_7W_1Co_{10}[H_2P_8W_{48}O_{186}]\cdot 132H_2O$
**1**$_{\mathbf{dehydrated}}$	Compound **1** evacuated for 30 minutes in water bath (80˚C)	0	1.05	$Li_9K_7W_1Co_{10}[H_2P_8W_{48}O_{186}]\cdot 74H_2O$
**2a**	Compound **1** put in NH_3 for 1 min	4.63	1.67	$Li_9K_7W_1Co_{10}[H_2P_8W_{48}O_{186}]\cdot 42H_2O\cdot 46NH_3$
**2b**	Compound **1** put in NH_3 for 30 min	4.23	1.45	$Li_9K_7W_1Co_{10}[H_2P_8W_{48}O_{186}]$ $35H_2O\cdot 44NH_3$
**2c**	Compound **1**$_{\mathbf{c}}$ put in NH_3 for 1 min	3.29	1.25	$Li_9K_7W_1Co_{10}[H_2P_8W_{48}O_{186}]\cdot 30H_2O\cdot 35NH_3$
**3**	Compound **1** put in CH_3OH vapour for 30 min	1.57	1.02	$Li_9K_7W_1Co_{10}[H_2P_8W_{48}O_{186}]$ $40H_2O\cdot 18CH_3OH$

Supplementary Table 2. Microanalysis of N, H of compound $\mathbf{2_a}$ in different conditions.

Compound	Description	N(%)	H(%)	Formula
$\mathbf{2a_{-vacuum}}$	Compound $\mathbf{2_a}$ put in vacuum for 30 min	3.56	1.33	$Li_9K_7W_1Co_{10}[H_2P_8W_{48}O_{186}]\cdot 35H_2O\cdot 37NH_3$
$\mathbf{2a_{-vacuum-NH3}}$	Compound $\mathbf{2_{a-vacuum}}$ put in NH_3 for 1 min	3.96	1.42	$Li_9K_7W_1Co_{10}[H_2P_8W_{48}O_{186}]\cdot 35H_2O\cdot 42NH_3$
$\mathbf{2a_{-air}}$	Compound $\mathbf{2_a}$ put in air for 1 day	0.97	1.05	$Li_9K_7W_1Co_{10}[H_2P_8W_{48}O_{186}]\cdot 45H_2O\cdot 10NH_3$
$\mathbf{2a_{-air-NH3}}$	Compound $\mathbf{2_{a-air}}$ put in NH_3 for 1 min	4.68	1.57	$Li_9K_7W_1Co_{10}[H_2P_8W_{48}O_{186}]\cdot 38H_2O\cdot 49NH_3$
$\mathbf{2a_{-steam}}$	Compound $\mathbf{2_a}$ put in steam for 5 min	0.79	0.76	$Li_9K_7W_1Co_{10}[H_2P_8W_{48}O_{186}]\cdot 38H_2O\cdot 8NH_3$
$\mathbf{2a_{-steam-NH3}}$	Compound $\mathbf{2_{a-steam}}$ put in NH_3 for 1 min	4.81	1.64	$Li_9K_7W_1Co_{10}[H_2P_8W_{48}O_{186}]\cdot 38H_2O\cdot 51NH_3$
$\mathbf{2a_{-humidifier}}$	Compound $\mathbf{2_a}$ put in humidifier for 1 day	0.18	1.78	$Li_9K_7W_1Co_{10}[H_2P_8W_{48}O_{186}]\cdot 128H_2O\cdot 2NH_3$
$\mathbf{2a_{-humidifier-1}}$	Compound $\mathbf{2_a}$ put in humidifier for 2 weeks	0	1.73	$Li_9K_7W_1Co_{10}[H_2P_8W_{48}O_{186}]\cdot 131H_2O$
$\mathbf{2a_{-humidifier-NH3}}$	Compound $\mathbf{2_{a-humidifier}}$ put in NH_3 for 1 min	4.98	1.76	$Li_9K_7W_1Co_{10}[H_2P_8W_{48}O_{186}]\cdot 42H_2O\cdot 53NH_3$

Supplementary Table 3. Microanalysis of N, H of compound **2_c** in different conditions.

Compound	Description	N(%)	H(%)	Formula
**$2c_{-vacuum}$**	Compound **2_c** put in vacuum for 30 min	3.16	1.11	$Li_9K_7W_1Co_{10}[H_2P_8W_{48}O_{186}]\cdot 30H_2O\cdot 32NH_3$
**$2c_{-vacuum-NH3}$**	Compound **$2_{c-vacuum}$** put in NH_3 for 1 min	3.44	1.11	$Li_9K_7W_1Co_{10}[H_2P_8W_{48}O_{186}]\cdot 20H_2O\cdot 35NH_3$
**$2c_{-air}$**	Compound **2_c** put in air for 1 day	1.30	0.88	$Li_9K_7W_1Co_{10}[H_2P_8W_{48}O_{186}]\cdot 38H_2O\cdot 13NH_3$
**$2c_{-air-NH3}$**	Compound **2_{c-air}** put in NH_3 for 1 min	4.34	1.44	$Li_9K_7W_1Co_{10}[H_2P_8W_{48}O_{186}]\cdot 32H_2O\cdot 46NH_3$
**$2c_{-steam}$**	Compound **2_c** put in steam for 5 min	0.72	0.79	$Li_9K_7W_1Co_{10}[H_2P_8W_{48}O_{186}]\cdot 40H_2O\cdot 7NH_3$
**$2c_{-steam-NH3}$**	Compound **$2_{c-steam}$** put in NH_3 for 1 min	4.26	1.54	$Li_9K_7W_1Co_{10}[H_2P_8W_{48}O_{186}]\cdot 38H_2O\cdot 45NH_3$
**$2c_{-humidifier}$**	Compound **2_c** put in humidifier for 1 day	0.17	1.52	$Li_9K_7W_1Co_{10}[H_2P_8W_{48}O_{186}]\cdot 106H_2O\cdot 2NH_3$
**$2c_{-humidifier-1}$**	Compound **2_c** put in humidifier for 2 weeks	0	1.53	$Li_9K_7W_1Co_{10}[H_2P_8W_{48}O_{186}]\cdot 112H_2O$
**$2c_{-humidifier-NH3}$**	Compound **$2_{c-humidifier}$** put in NH_3 for 1 min	4.72	1.67	$Li_9K_7W_1Co_{10}[H_2P_8W_{48}O_{186}]\cdot 42H_2O\cdot 50NH_3$

Supplementary Table 4. ICP-OES data for parent compound **1**.

Element	%	Theoretical (%)
Co	3.91	3.79
K	2.08	1.76
Li	0.55	0.4
P	1.54	1.59
W	59.81	57.98

Supplementary Table 5. Crystallographic details

	**1**	$\mathbf{1}_{\mathbf{rehydrated}}$	$\mathbf{1}_{\mathbf{part.rehyd.}}$
Empirical formula	$H_{266}Co_{10}K_7Li_9O_{318}P_8W$	$H_{252}Co_{10}K_7Li_9O_{311}P_8W$	$H_{192}Co_{10}K_7Li_9O_{281}P_8W$
CCDC	1429443	1429446	1429445
F_w (g mol^{-1})	15537.98	15411.87	14871.39
Wavelength (Å)	0.71073 (Mo K_α)	0.71073 (Mo K_α)	0.71073 (Mo K_α)
Crystal size (mm)	0.28 x 0.24 x 0.13	0.10 x 0.07 x 0.04	0.23 x 0.14 x 0.14
Crystal system	Triclinic	Triclinic	Triclinic
Space group	*P*-1	*P*-1	*P*-1
a (Å)	13.8970(2)	13.4268(9)	11.2609(3)
b (Å)	22.9151(3)	22.7936(16)	21.8187(7)
c (Å)	24.5947(4)	24.5244(17)	22.1523(7)
α (°)	64.430(2)	64.136(4)	70.700(3)
β (°)	85.146(2)	77.827(4)	86.570(2)
γ (°)	88.117(2)	87.681(8)	82.208(2)
V (Å^3)	7039.72(18)	6590.6(8)	5088.8(3)
Z	1	1	1
ρ_{calcd} (g cm^{-3})	3.665	3.883	4.853
μ (mm^{-1})	20.777	22.190	28.718
T (K)	150(2)	150(2)	150(2)
Absorption correction	Analytical	Empirical	Analytical
Goodness-of-fit on F^2	1.047	1.094	0.880
No. of reflections	108953	57665	56220
No. of reflections	26642	20966	16033
R_{int}	0.0438	0.0870	0.0721
Residuals: $R_{1(obs)}$; ${}_{w}R_{2(all}$	0.0381; 0.1064	0.0830; 0.2461	0.0426; 0.0916

Supplementary Table 5. Crystallographic details (continued)

	**1**$_{\mathbf{dehydrated}}$	**2a**	**2b**
Empirical formula	$H_{60}Co_{10}K_7Li_9O_{215}P_8W$	$H_{224}Co_{10}K_7Li_9N_{46}O_{228}P_8$	$H_{204}Co_{10}K_7Li_9N_{44}O_{221}P_8$
CCDC	1429444	1429447	1429449
F_w (g mol^{-1})	14493.06	14700.11	14539.93
Wavelength (Å)	0.71073 (Mo K_α)	0.71073 (Mo K_α)	0.71073 (Mo K_α)
Crystal size (mm)	0.28 x 0.23 x 0.14	0.09 x 0.06 x 0.04	0.11 x 0.08 x 0.04
Crystal system	Triclinic	Triclinic	Triclinic
Space group	*P*-1	*P*-1	*P*-1
a (Å)	11.2207(4)	13.5401(7)	13.5578(5)
b (Å)	21.2571(8)	22.4119(10)	21.7073(9)
c (Å)	22.0231(8)	24.2578(11)	25.0497(11)
α (°)	68.713(3)	64.697(2)	73.005(3)
β (°)	87.051(3)	77.745(2)	76.461(2)
γ (°)	84.305(3)	88.032(2)	83.344(2)
V (Å^3)	4869.7(3)	6490.1(5)	6845.4(5)
Z	1	1	1
ρ_{calcd} (g cm^{-3})	4.942	3.761	3.527
μ (mm^{-1})	29.995	22.505	21.333
T (K)	150(2)	150(2)	150(2)
Absorption correction	Analytical	Empirical	Empirical
Goodness-of-fit on F^2	1.128	1.007	1.117
No. of reflections (measured)	67159	81288	71621
No. of reflections	16937	22135	22633
R_{int}	0.0847	0.0731	0.1048
Residuals: $R_{1(obs)}$; $_{w}R_{2(all\ data)}$	0.1481; 0.3525	0.0593; 0.1839	0.0757; 0.1952

Supplementary Table 5. Crystallographic details (continued)

	**2c**	**$2a_{rehydrated}$**	**3**
Empirical formula	$H_{167}Co_{10}K_7Li_9N_{35}O_{216}P_8$	$H_{122}Co_{10}K_7Li_9N_{10}O_{231}P$	$C_{18}H_{154}Co_{10}K_7Li_9O_{244}P$
CCDC	1429450	1429448	1429451
F_w (g mol^{-1})	14296.55	14140.94	14457.27
Wavelength (Å)	0.71073 (Mo K_{α})	0.71073 (Mo K_{α})	0.71073 (Mo K_{α})
Crystal size (mm)	0.10 x 0.08 x 0.03	0.10 x 0.06 x 0.05	0.10 x 0.09 x 0.08
Crystal system	Triclinic	Triclinic	Triclinic
Space group	*P*-1	*P*-1	*P*-1
a (Å)	13.7671(8)	13.7940(8)	14.0297(10)
b (Å)	22.1476(13)	22.9102(13)	23.2717(15)
c (Å)	24.542(2)	24.4981(14)	23.9190(16)
α (°)	114.650(6)	64.310(3)	65.981(4)
β (°)	103.065(6)	85.448(3)	89.266(4)
γ (°)	96.884(4)	89.983(4)	85.046(4)
V (Å^3)	6428.3(8)	6949.7(7)	7104.4(9)
Z	1	1	1
ρ_{calcd} (g cm^{-3})	3.693	3.379	3.379
μ (mm^{-1})	22.711	21.007	20.556
T (K)	150(2)	150(2)	150(2)
Absorption correction	Empirical	Empirical	Empirical
Goodness-of-fit on F^2	1.007	1.064	1.083
No. of reflections (measured)	61976	36220	97775
No. of reflections	23187	36220	27857
R_{int}	0.1986	0.0661	0.0879
Residuals: $R_{1(obs)}$;	0.0850; 0.1644	0.0661; 0.1891	0.0697; 0.2141
